# Structural basis of ribosomal RNA transcription regulation

**DOI:** 10.1038/s41467-020-20776-y

**Published:** 2021-01-22

**Authors:** Yeonoh Shin, M. Zuhaib Qayyum, Danil Pupov, Daria Esyunina, Andrey Kulbachinskiy, Katsuhiko S. Murakami

**Affiliations:** 1 Department of Biochemistry and Molecular Biology, Pennsylvania State University, University Park, PA 16802 USA; 2 Institute of Molecular Genetics, Russian Academy of Sciences, Moscow, 123182 Russia

**Keywords:** Holoenzymes, Bacterial transcription, Transcriptional regulatory elements, Cryoelectron microscopy

## Abstract

Ribosomal RNA (rRNA) is most highly expressed in rapidly growing bacteria and is drastically downregulated under stress conditions by the global transcriptional regulator DksA and the alarmone ppGpp. Here, we determined cryo-electron microscopy structures of the *Escherichia coli* RNA polymerase (RNAP) σ^70^ holoenzyme during rRNA promoter recognition with and without DksA/ppGpp. RNAP contacts the UP element using dimerized α subunit carboxyl-terminal domains and scrunches the template DNA with the σ finger and β’ lid to select the transcription start site favorable for rapid promoter escape. Promoter binding induces conformational change of σ domain 2 that opens a gate for DNA loading and ejects σ_1.1_ from the RNAP cleft to facilitate open complex formation. DksA/ppGpp binding also opens the DNA loading gate, which is not coupled to σ_1.1_ ejection and impedes open complex formation. These results provide a molecular basis for the exceptionally active rRNA transcription and its vulnerability to DksA/ppGpp.

## Abstract (web summary)

Ribosomal RNA (rRNA) expression is regulated at the initiation stage of RNA synthesis. Here, the authors report cryo-EM structures of *E. coli* RNA polymerase and rRNA promoter complex with DksA/ppGpp on the way to open complex formation, identifying key steps in promoter recognition and opening.

## Introduction

Bacteria sense the availability of nutrition and adjust ribosome biogenesis to optimize their growth. The rate of ribosome biogenesis is primarily determined by rRNA transcription^1,2^, which constitutes as much as 70% of total RNA synthesis and is initiated approximately every second from each of the seven rRNA operons (*rrn*A-E and *rrn*G-H) in *E. coli* during exponential growth^3^. However, it is drastically repressed under stress conditions such as nutrient-starved stationary phase^4^. rRNA expression is primarily regulated at the initiation stage of RNA synthesis, including RNAP binding to promoter DNA, unwinding the DNA, and escaping from the promoter.

**Fig. 1:**
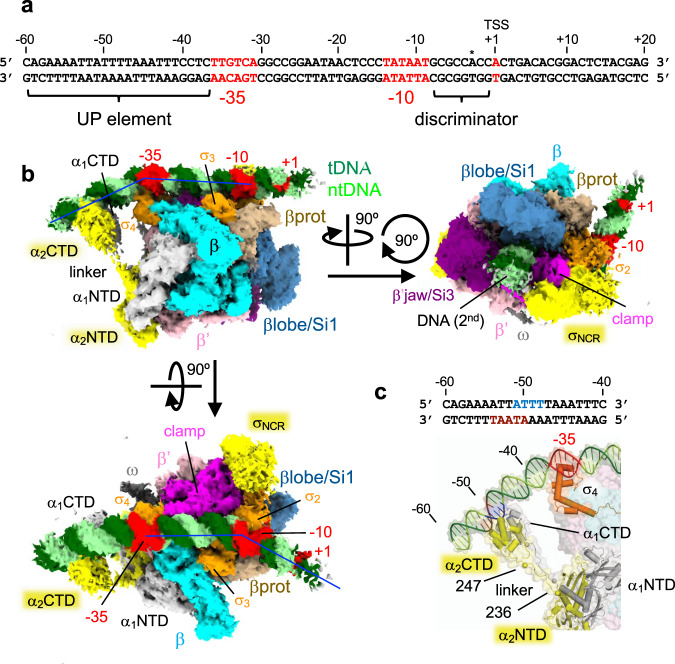
Cryo-EM structure of the RNAP-*rrnB*P1 closed complex (RPc). **a** The sequence of the *E. coli rrnB*P1 promoter DNA used for cryo-EM. The UP element, −35 element, −10 element, transcription start site (TSS, + 1) and discriminator sequence are indicated. Alternative TSS from the nonscrunched open complex is indicated by an asterisk. **b** Orthogonal views of the RPc cryo-EM density map. Subunits and domains of RNAP and DNA are colored and labeled (βprot, βprotrusion; tDNA, template DNA; ntDNA, nontemplate DNA). The density of downstream DNA beyond the +4 position is not traceable. Blue lines denote the direction of the DNA axis, with kinks at ~−37 and −13. The second DNA at the RNAP cleft is indicated (DNA (2nd)). **c** A magnified view showing the αCTDs and UP element interaction. The domains of α subunits, σ_4_, and DNA are depicted as ribbon models with a partially transparent surface. At the top, the sequence of the UP element is shown. The ntDNA (−51 to −48) and tDNA (−54 to −50) sequences binding α_1_CTD and α_2_CTD are highlighted in blue and brown, respectively.

The promoters (e.g., *rrnB*P1) for expressing rRNA operons are unique compared with other σ^70^-dependent promoters, including (1) the A + T-rich UP element located upstream of the −35 element (from −60 to −40); (2) the G + C-rich discriminator sequence downstream of the −10 element (from −8 to −1); and (3) the transcription start site (TSS) located nine bases downstream from the −10 element (Fig. 1a and Supplementary Fig. 1a). The UP element is recognized by the carboxyl-terminal domain of the α subunit (αCTD) and enhances rRNA transcription by more than 30-fold^5^. The G + C-rich discriminator and unusual TSS selection of rRNA promoters make its open complex (RPo) unstable, but facilitate RNAP escape from the promoter by reducing abortive RNA cycle prior to the RNA elongation stage^6^. These promoter elements play key roles in the wide range of rRNA transcription regulation between nutrient-rich and -poor growth conditions.

rRNA transcription activity is regulated by two small molecules—the initiating ribonucleotide (iNTP) (ATP in the case of *rrnB*P1)^7^ and the bacterial alarmone ppGpp (guanosine tetraphosphate, aka “magic spot”), which is an allosteric effector of the RNAP-binding global transcription regulator DksA^8–10^. In the presence of high iNTP concentration, rRNA synthesis starts immediately after RNAP formed the RPo on rRNA promoters, allowing rapid transition to transcription elongation (promoter escape). However, the iNTP-limited condition shifts the equilibrium to favor early intermediates in promoter complex formation, including the closed complex, which is further shifted  by DksA/ppGpp binding to RNAP^4^. The ppGpp concentration is increased under stress conditions, which enhances DksA-mediated rRNA repression by stabilizing RNAP–DksA complex in a functionally important binding mode^11^.

The majority of bacterial RNAP–DNA complex structures determined by X-ray crystallography contain short promoter DNA fragments with a premelted transcription bubble that mimics RPo to maximize its stability required for time-consuming crystallization method^12,13^. These studies explained the structural basis of promoter recognition and transcript initiation but left unexplored the interactions of RNAP with duplex DNA around the UP element (via αCTDs) and the contacts with the −10 element (via σ domain 2, residues 92–127 and 373–456 in σ^70^) in a closed complex and the scrunched DNA bubble in the RPo formed with the rRNA promoters.

Cryo-electron microscopy (cryo-EM) structures of the *E. coli* RNAP-*rpsT*P2 promoter complex with a ppGpp-insensitive DksA homolog TraR revealed the RPo formation pathway in the presence of TraR^14^. However, the *rpsT*P2 promoter for expressing ribosomal protein S20 is distinct from the *rrnB*P1 promoter that it contains G + C-rich DNA upstream of the −35 element and the TSS 7 bases downstream from the −10 element; therefore, it does not reveal the pathway for rRNA promoter complex formation and the mechanism of rRNA transcription regulation. In addition, the presence of TraR does not allow to infer the unperturbed pathway of the open complex formation by RNAP^15–17^. Here, we used cryo-EM to visualize the RNAP and *rrnB*P1 complexes and two additional complexes with DksA/ppGpp on the way to RPo formation.

## Results

### Cryo-EM structure of the RNAP and *rrnB*P1 promoter closed complex

To obtain promoter complexes of RNAP with *rrnB*P1, we preincubated *E. coli* RNAP σ^70^ holoenzyme with promoter DNA (Fig. 1a) at 37 °C for 5 min; as a possible way to stabilize the complex, we also added NTPs (ATP and the nonhydrolyzable CTP analog CMPCPP for +1 and +2 NTPs, respectively) prior to cryo-EM grid preparation. In the course of cryo-EM data processing, 3D classification revealed two distinct structures (“Methods” and Supplementary Fig. 2), corresponding to a closed promoter complex (here designated RPc) and the transcript initiation complex (RPtic) containing 2-mer RNA. In the RPtic, the 2-mer RNA transcript (5′-CpA-3′) base-pairs with −1G and +1T in template DNA and is positioned in the post-translocated conformation. The detailed RPtic structure will be described in a separate report.

We determined the RNAP-*rrnB*P1 RPc structure with an overall resolution of 4.14 Å (Supplementary Table 1). The cryo-EM density shows that RNAP binds the duplex DNA from −60 to +3, which remains fully double-stranded (Fig. 1b, Supplementary Fig. 4 and Supplementary Movie 1), but the density of downstream DNA beyond position +4 is not traceable. Instead, a second DNA binds to the RNAP cleft due to ejection of σ_1.1_ from the RNAP cleft during RPc formation as described later.

The cryo-EM density for both αCTDs (residues 248–329), the linkers (residues 236–247) connecting to αNTDs (residues 1–235), and the UP element DNA were traceable in the RPc, allowing us to investigate how each αCTD binds to the UP element unambiguously (Fig. 1c and Supplementary Movie 1). Two αCTDs form a head-to-tail dimer and bind DNA side-by-side in the middle of the UP element (−51 to −48 on nontemplate DNA (ntDNA) and −54 to −50 on template DNA (tDNA)), which is in good agreement with the DNA footprinting results^18^. Although α subunits form a homodimer, two α subunits play different roles in RNAP, with one (α_1_) adjacent to the β subunit and the other (α_2_) adjacent to the β’. Compared to the α_2_CTD, the α_1_CTD is positioned proximally to the −35 element, which explains the result of DNA cleavage by hydroxyl radicals from chelated Fe attached at each of the two αCTDs^19^. The αCTDs bind DNA with a narrow minor groove, which is formed due to the presence of an A/T stretch sequence, as revealed by the recent X-ray crystallographic study of the αCTD and UP element interaction^20^. The side chains of R265 and N294 from both αCTDs are inserted into the DNA minor groove, and basic residues (K291 and K298) are involved in salt bridges with the DNA phosphate backbone (Supplementary Fig. 5a). The linkers of both α subunits are fully extended, and slight DNA bending centered at the −37 position is required for the αCTDs binding to the UP element (Fig. 1c). Consistent with this observation, shortening of the linkers by only three amino acids reduces *rrnB*P1 transcription^21^. Several studies have proposed that distant upstream DNA (near the −100 position) warps around RNAP on the RPo formation pathway, and the interaction of αCTDs and the UP element is one of the major driving forces for this DNA wrapping^22,23^. However, αCTDs do not bend the DNA around their binding site in the RPc structure determined in this study. This observation suggests that the contacts of αCTDs with the UP element by themselves are not sufficient for wrapping of upstream DNA around RNAP. This may possibly result from the destabilization of upstream DNA and RNAP interactions by truncation of the promoter fragment at the −60 position and/or from specific conditions used for the cryo-EM sample preparation.

The position and orientation of αCTDs in the *rrnB*P1 RPc structure are distinct from those of αCTDs in the RNAP complex with the *rpsT*P2 promoter, lacking the UP element (PDB: 6PSQ)^14^, which binds DNA just upstream of the σ domain 4 (σ_4_) bound at the −35 element (Supplementary Fig. 5b). This indicates that the mode of αCTDs interactions with upstream DNA can be significantly different in various promoters depending on the presence of the UP element.

The RPc structure shows how σ_2_ binds the duplex form of the −10 element. The DNA encoding the −10 element is anchored by σ domain 2 and slightly bends around the upstream edge of the −10 element, allowing the downstream part beyond the −10 element to reach the other side of the RNAP cleft comprising the β protrusion domain (Fig. 1b and Supplementary Movie 1). The σ region 2.3 (σ_2.3_, residues 417–434) contacts the −10 element by fitting into the DNA major groove seemingly without sequence-specific interaction, indicating that σ_2.3_ recognizes the shape and/or curvature around the −10 element. This finding is in agreement with the previous proposal^24^ that σ^70^ does not contact the −10 element DNA bases when it is in duplex form.

### Cryo-EM structure of the RNAP and *rrnB*P1 promoter open complex (RPo)

**Fig. 2:**
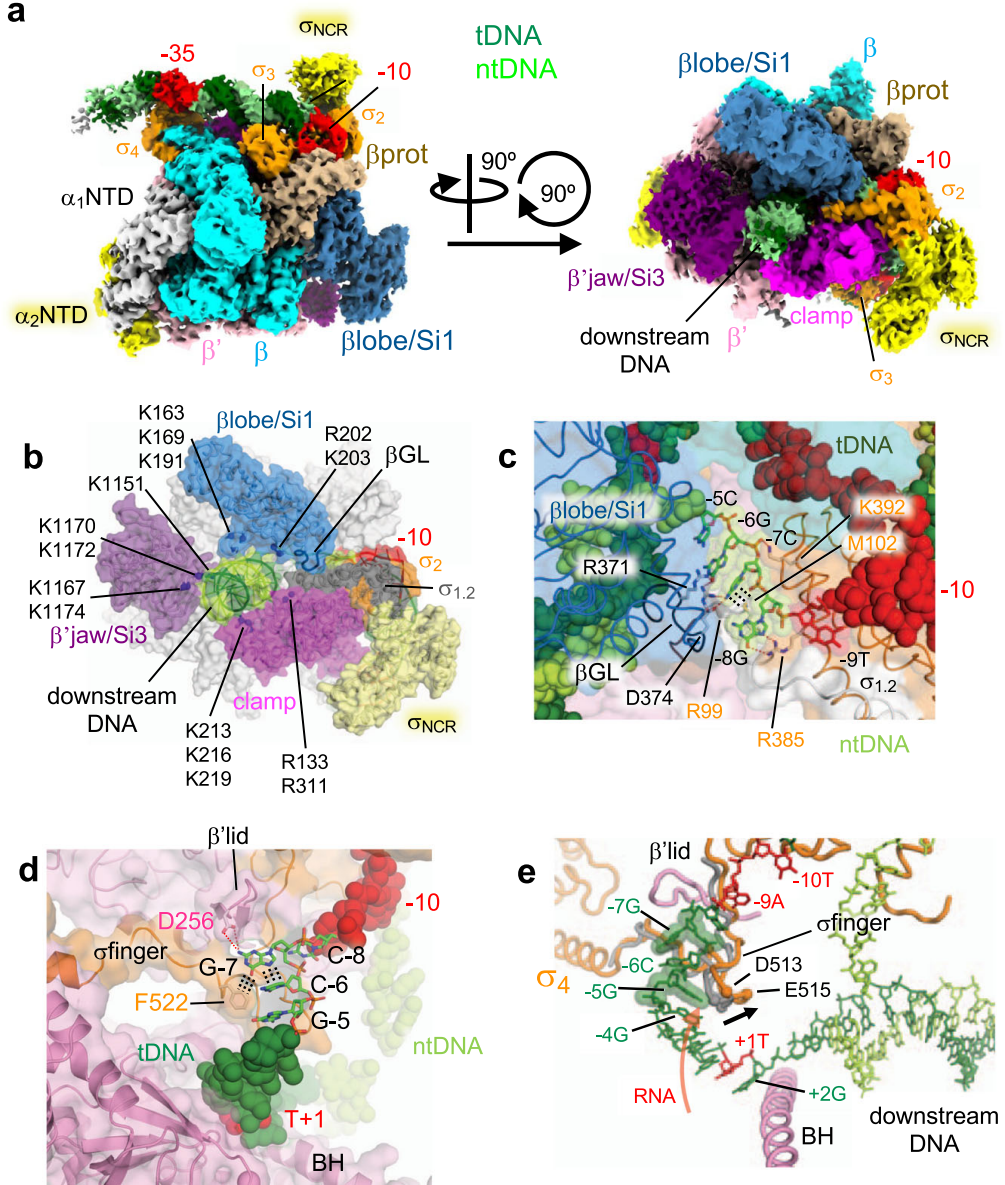
Cryo-EM structure of the RNAP-*rrnB*P1 open complex (RPo). **a** Orthogonal views of the RPo cryo-EM density map. Subunits and domains of RNAP and DNA are colored and labeled the same as in Fig. 1b. **b** The structure of the RPo, highlighting basic residues of the βlobe/Si1 (blue), β’jaw/Si3 (purple), and β’clamp (pink) interacting with downstream DNA (green) to stabilize the RPo. The structure is shown as a ribbon model with a transparent surface, and the basic residues are shown as spheres and labeled. **c** Close-up view of RNAP (βGL, σ_1.1_, and σ_1.2_) and discriminator DNA (ntDNA) interaction. β and σ are depicted as ribbon models with transparent surfaces, and DNA is shown as CPK spheres. The G-8, C-7, and G-6 bases (stick model with transparent CPK spheres) that form salt bridges and Van del Waals interactions with residues from the βGL and σ_1.2_ (side chains shown as sticks; βGL R371 and D374; σ_1.2_ R99 and M102) are shown (depicted by red and black dashed lines). **d** Close-up view of the RNAP (β’lid and σfinger) and discriminator DNA (tDNA) interaction. The G-7 base inserts into the pocket formed by the β’lid, σfinger and C-6 base. β’ and σ are depicted as ribbon models with transparent surfaces, and DNA is shown as a stick model and CPK representation. The residues forming salt bridges and Van del Waals interactions with the G-7 base are shown (depicted by red and black dashed lines). **e** Comparison of the σfinger in RPo-*rrnB*P1 (this study, orange) and RPo-*rpsT*P2^14^ (gray). The RPo-*rrnB*P1 structure is depicted as a cartoon (RNAP) and stick (DNA) models. When the *rrnB*P1 tDNA scrunches at the −7G(t) position, −5G(t) is located below the σfinger (orange), which shifts the σfinger position compared to that in nonscrunched RPo (gray). The σfinger dislocation (black arrow, 5 Å at E515) makes additional space for RNA extension (red arrow).

To obtain the structure of the open RNAP-*rrnB*P1 complex (RPo) with melted DNA bubble, we preincubated RNAP holoenzyme and promoter DNA at 37 °C for 5 min prior to cryo-EM grid preparation (Supplementary Fig. 3). 3D classification revealed one major class of particles corresponding to RPo, and its structure was determined with an overall resolution of 3.5 Å. In comparison with the RPc, the RPo structure shows significant differences in the UP element (from −60 to −40), the downstream DNA (from −14 to +20) and the conformation of the σ factor. The cryo-EM density for RPo covers DNA from −44 to +20, including an open bubble from −13 to +2 and the downstream DNA accommodated in the RNAP cleft (Fig. 2a, Supplementary Fig. 4b,e, and Supplementary Movie 2). In contrast to the RPc, αCTDs and UP elements are disordered.

Basic residues in the βlobe (K163, K169, K191, R202, and K203), β’jaw (K1151, K1167, K1170, K1172, and R1174) and β’clamp (R133, K213, K216, K219, and R311) participate in the interaction with downstream DNA to stabilize the RPo (Fig. 2b). The importance of these interactions in rRNA transcription regulation is supported by the isolation of Δ*dksA* suppressor mutations in these domains^25,26^. The βgate loop (βGL, residues 368–378) in the βlobe domain contacts the T94, R99, and R103 residues of σ region 1.2 (σ_1.2_, residues 92–127) to enclose the RNAP cleft (Fig. 2b). The βGL, σ_1.2_, and σ_2.1_ (residues 373–396) contact the ntDNA strand of the discriminator from positions −8 to −6 (Fig. 2c and Supplementary Movie 3); consistently, the βGL deletion and substitutions in σ_1.2_ destabilize the RPo^27,28^.

**Fig. 3:**
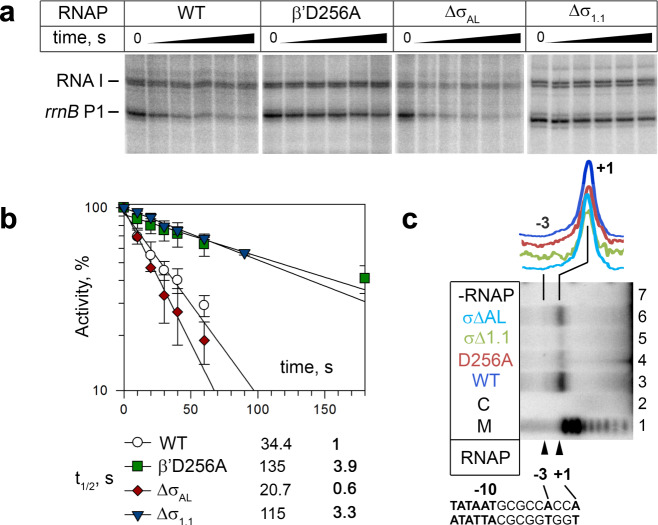
Stabilities of *rrnB*P1 promoter complexes formed by wild-type and mutant RNAPs and their sensitivities against DksA/ppGpp. **a** Stabilities of *rrnB*P1 promoter complexes. Preformed complexes of *E. coli* RNAP with supercoiled plasmid DNA containing the *rrnB*P1 and RNAI promoters were incubated with competitor DNA (containing upstream fork-junction promoter DNA  but lacking the TSS) for the indicated time intervals, followed by the addition of NTP substrates. Positions of the full-length transcripts are indicated. For each RNAP, the experiments were repeated independently two to five times with similar results. **b** Kinetics of promoter complex dissociation for wild-type and mutant RNAPs. The half-lives of the promoter complexes for each RNAP are shown (measured in the absence of DksA; mean values from two to five independent experiments). **c** Transcription start site mapping in *rrnB*P1 for wild-type and mutant RNAPs (a representative gel from two independent experiments). The products of in vitro transcription from *rrnB*P1 were used for primer extension with a 5′-labeled downstream primer, and the lengths of the products were compared with the starting primer (lane 2) and size markers (lane 1). Lane 7 shows a control reaction performed in the absence of RNAP. Positions of the transcription start sites in the scrunched (+1) and relaxed (−3) promoter complexes are indicated. The scanned profiles of the primer extension products adjusted by the intensity of the main product for each RNAP are shown above the gel. Source data are provided as a Source Data file.

The RNAP and *rrnB*P1 complex starts RNA synthesis at the position 9 bp downstream from the −10 element (+1A), which requires DNA scrunching^29^. The RPo structure revealed the path of the scrunched template strand, in which the G-7 base of tDNA fits into a pocket surrounded by the β’lid, σfinger (σ region 3.2) and the C-6 base (Fig. 2d, Supplementary Fig. 4b, e and Supplementary Movie 3). The importance of the G-7 base for rRNA expression is underscored by its conservation in all seven rRNA promoters in *E. coli* and rRNA promoters in other proteobacteria (Supplementary Fig. 1). Highly conserved D256 (β’lid) and F522 (σfinger) residues form a salt bridge and Van der Waals interaction with the G-7 base, respectively. We found that alanine substitution of residue D256 (β’lid) significantly stabilizes RNAP complexes with the *rrnB*P1 promoter (promoter complex half-life t_1/2_ of 135 ± 16 s vs. 34 ± 7 s for wild-type RNAP) (Fig. 3a, b). Similarly, a σ finger deletion was shown to increase the stability of RNAP-*rrnB*P1 complex^30^, suggesting that the contacts of σfinger with tDNA may destabilize this promoter complex. The deletion of the σ finger^30^ or the G-7C substitution^29^ was also shown to shift the TSS to the −3A position, likely eliminating open complex scrunching. However, the D256A substitution did not change the TSS in *rrnB*P1, as revealed by primer extension analysis of the transcription products synthesized from *rrnB*P1 by wild-type and mutant RNAPs (Fig. 3c, lanes 3 and 4). Therefore, the contacts of the β’lid with G-7 may decrease the stability of the RNAP-*rrnB*P1 complex with scrunched DNA, possibly by favoring the unstable conformation of the template strand, without changing the TSS selection. The nontemplate DNA strand is also scrunched, making DNA bases around the single-stranded and double-stranded junction at the RNAP active site (from −2 to +2 positions) disordered.

Open complex scrunching may facilitate promoter escape of RNAP by reducing abortive RNA cycle^29,31^. Compared with RPo-*rpsT*P2 containing nonscrunched tDNA^14^, RPo-*rrnB*P1 shifts the σfinger ~5 Å away from tDNA, allowing accommodation of one additional base of RNA before its 5′-end reaches the σfinger (Fig. 2e). Since the σfinger is one of the major obstacles to promoter escape^32–34^, the partially displaced σfinger in the RPo may reduce the abortive RNA cycle or may prevent the formation of inactive moribund complexes^31,35^, promoting the robust expression of rRNA.

### Cryo-EM structures of the RNAP and *rrnB*P1 promoter complex with DksA/ppGpp (RP-DksA/ppGpp)

**Fig. 4:**
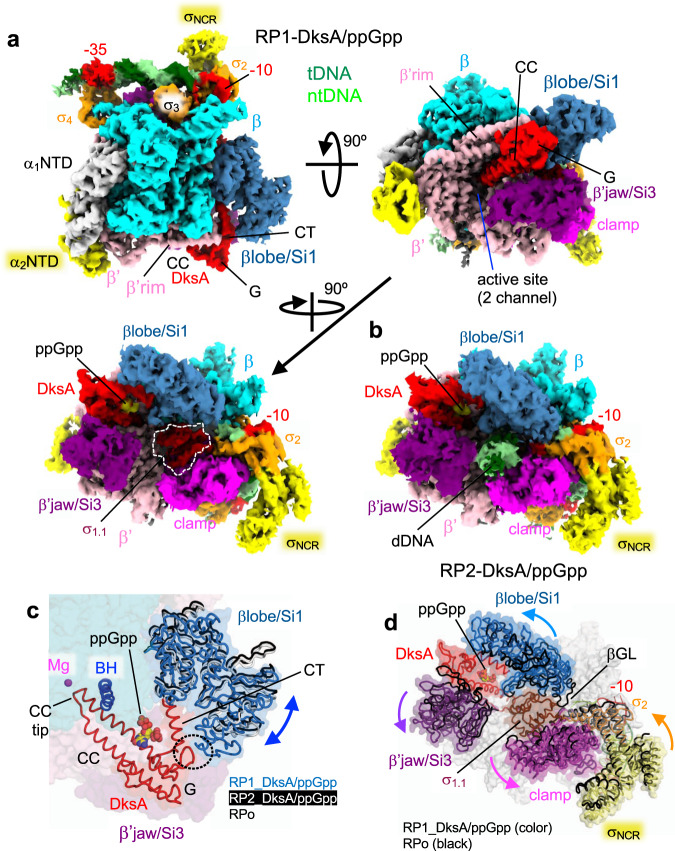
Cryo-EM structures of the RNAP-*rrnB*P1 complex with DksA/ppGpp (RP-DksA/ppGpp). **a** Orthogonal views of the RP1-DksA/ppGpp cryo-EM density map. DNA, RNAP and DksA (G, G domain; CC, CC domain; CT, CT-helix) are indicated and colored. σ_1.1_ is highlighted by a white dash. **b** The RP2-DksA/ppGpp cryo-EM density map. The downstream DNA is accommodated in the RNAP cleft. **c** Close-up view of the βlobe/Si1 conformational changes upon DksA binding, σ_1.1_ ejection and downstream DNA binding. The structures of the βlobe/Si1 in RP1-DksA/ppGpp (light blue), RP2-DksA/ppGpp (white), and RPo (black) are depicted as ribbon models with transparent surfaces and ribbon models (DksA, BH: bridge helix) of RP1-DksA/ppGpp. The interaction between βlobe/Si1 and DksA in RP1-DksA/ppGpp is highlighted by a black dashed oval. **d** Conformational changes in the RNAP mobile domains upon binding of DksA/ppGpp. The structures of the RNAP mobile domains in RP1-DksA/ppGpp (colored) and RPo (black) are depicted as ribbon models with transparent surfaces of RP1-DksA/ppGpp.

To reveal how DksA/ppGpp binding to RNAP downregulates rRNA transcription, we visualized the RNAP, *rrnB*P1, and DksA/ppGpp complex (RP-DksA/ppGpp) by cryo-EM (Supplementary Table 1 and Supplementary Fig. 6). The classification of the cryo-EM data gave rise to two structures that differed mainly within the RNAP cleft; the first class shows the globular density corresponding to σ_1.1_ (class I, RP1-DksA/ppGpp), and the second class shows the helical density corresponding to the downstream DNA (class II, RP2-DksA/ppGpp) (Fig. 4a, b and Supplementary Movie 4). In addition, the positions of βlobe/Si1 are different in these classes (Fig. 4c).

 In both classes, the cryo-EM density maps show  ppGpp binding at sites 1 and 2 and DksA binding at the RNAP secondary channel (Supplementary Fig. 6e), as observed in the previous X-ray crystallography study^11^. DksA binds RNAP with its globular domain (G domain, contacts with the β’rim helix), coiled-coil tip (CC tip, contacts with the active site), CC (contacts with the bridge helix, the trigger loop, and linkers connecting to the β’Si3), and the C-terminal α helix (CT-helix, contacts with the β lobe/SI1 domain) (Fig. 4a and Supplementary Movie 4). The CC of DksA prevents trigger helix formation and blocks NTP entry from the secondary channel, indicating that DksA must be displaced before RNAP initiates RNA synthesis^11,36^.

Both classes show the duplex DNA density from positions −42 to −14 (from the downstream edge of the UP element to the upstream edge of the −10 element) and also show the ssDNA density of the nontemplate strand of the −10 element (Supplementary Fig. 4). RP1-DksA/ppGpp retains σ_1.1_ in the RNAP cleft, indicating that it represents an early stage intermediate during the closed to open complexes transition. While the transcription bubble is likely partially open in RP1-DksA/ppGpp, the density of ntDNA from −5 to +20 and of tDNA from −13 to +20 is not traceable. Analysis of RP1-DksA/ppGpp reveals a DksA/ppGpp-induced conformational change in βlobe/Si1, β’jaw/Si3 and β’clamp, opening the downstream DNA cleft in RNAP and likely reducing the stability of RPo (Fig. 4d). The interactions of the βlobe Si1 domain with DksA CT-helix and the conformational change of Si1 were previously observed in the crystal structure of the RNAP–DksA/ppGpp complex^11^ but were smaller than in the cryo-EM structure, likely because of the crystal packing. The conformational change in βlobe/Si1 establishes a new contact with the DksA CT-helix, which is only observed in the cryo-EM structure (Fig. 4c and Supplementary Movie 4); the deletion of βSi1 was shown to reduce the DksA affinity to RNAP and impair its function^37^. Alanine substitution of an aspartate residue in the CT-helix directly involved in this interaction (D137A) decreases *rrnB*P1 inhibition by DksA both in the absence and in the presence of ppGpp (Table 1).

**Table 1:** Apparent affinities of DksA to wild-type and mutant RNAPs on the *rrnB*P1 promoter.

RNAP	*K*_d,app_ (nM)	
	−ppGpp	^+^ppGpp
WT	2200 ± 430	860 ± 180
	1	1
β′D256A	4540 ± 150	1640 ± 220
	2.1	1.9
Δσ_AL_	3000	1150 ± 220
	1.4	1.3
Δσ_1.1_	>10000	ND
WT RNAP +	3900	2440 ± 1100
DksA D137A	1.8	2.8

The apparent dissociation constants for DksA binding to promoter complexes (*K*_d,app_) were calculated from the efficiency of transcription inhibition in titration experiments. The numbers in below *K*_d,app_ values indicate fold changes in *K*_d,app_ relative to the wild-type RNAP.

The RP2-DksA/ppGpp complex contains downstream DNA (from +3 to +20) within the RNAP cleft, but the density of the DNA bubble (from −8 to +2) is not traceable (Fig. 4b and Supplementary Fig. 4), suggesting that it represents a late-stage intermediate before forming the RPo. The σ_1.1_ density is not traceable due to its ejection from the RNAP cleft. The conformations of βlobe/Si1 and β’clamp are akin to the RPo conformation, and the CT-helix of DksA does not contact with the βlobe/Si1 (Fig. 4c). Therefore, the transition between the two complexes may reduce the DksA affinity to RNAP and trigger its dissociation, which is an obligatory process to initiate RNA synthesis^11,36^.

### Ejection of σ_1.1_ and conformational changes in σ domain 2 during a RPc formation

The RPc structure revealed the ejection of σ_1.1_ from the RNAP cleft and significant conformational changes in σ domain 2, including σ_1.2_ and the nonconserved region between regions 1 and 2 (σ_NCR_, residues 128–372), in comparison with the apo-form holoenzyme RNAP^38^ and the RPo containing *rrnB*P1 (this study).

**Fig. 5:**
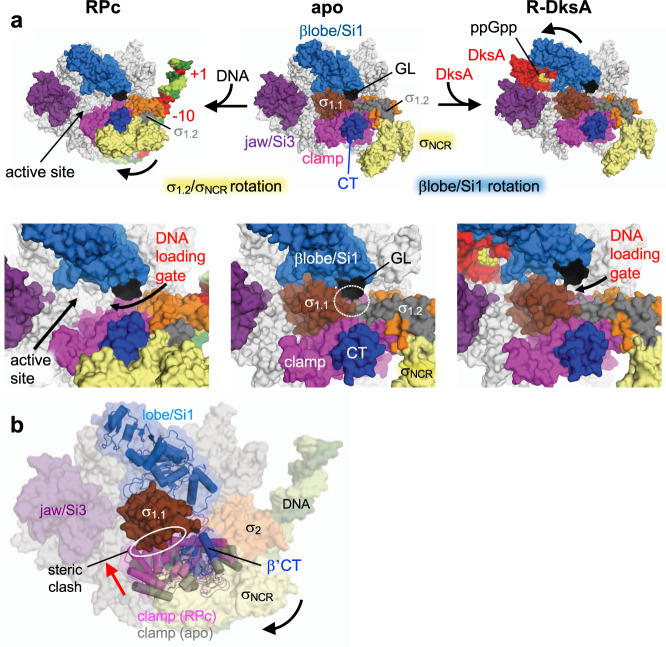
Opening the DNA loading gate by moving the σ_1.2_/σ_NCR_ or βlobe/Si1 domain. **a** Comparison of the σ_1.2_/σ_NCR_ and βlobe/Si1 conformations in apo-RNAP (middle), RPc (left), and RNAP-DksA/ppGpp (R-DksA, right, DNA is removed from the RP1-DksA/ppGpp to model this complex). RNAP (subunits and domains), DksA and DNA are indicated. Close-up views of the RNAP cleft are shown below. The DNA loading gate is closed in the apo-RNAP due to the βgate loop (βGL) contacts with σ_1.1_/σ_1.2_ (white dashed oval). The opening of the DNA loading gate in RPc and RP1-DksA/ppGpp is indicated by blue and red arrows, respectively. **b** A proposed model of σ_1.1_ ejection in the RPc. The RPc is depicted as a transparent surface with cartoon models of the clamp (purple) and the lobe/Si1 (blue). The clamp in the apo-form RNAP and the lobe/Si1 in RP1-DksA/ppGpp are colored gray and white, respectively. In the RPc, the σ_NCR_ rotation (black arrow) contacts the β’CT, resulting in clamp movement toward σ_1.1_ (red arrow) and a steric clash with σ_1.1_ (white oval).

From the apo-form to RPc, σ_1.2_/σ_NCR_ of RNAP holoenzyme undergo a rigid rotation toward the clamp to establish contact with the β’clamp-toe (β’CT, residues 143 to 180) (RPc, Fig. 5a). Although this interaction was not observed in any previous structural study, it was predicted based on the biochemical/genetic analysis of RNAP promoter escape and early elongation pausing^39^. It was shown that the interaction of the σ_NCR_ and β’CT is important for promoter escape and hinders early elongation pausing, and amino acid substitutions at the interface modulate both processes (Supplementary Fig. 7).

The βGL contacts the N-terminus of σ_1.2_ to enclose the RNAP cleft in the apo-form RNAP, which prevents DNA loading (apo, Fig. 5a), but the same interaction in the RPo stabilizes the open complex bubble (Fig. 2b). In the case of RPc, the σ_1.2_/σ_NCR_ rotation disrupts the βGL and σ contact and widens the gap that allows the ejection of σ_1.1_ and the entrance of discriminator DNA for the open complex bubble formation (RPc, Fig. 5a). Compared with the apo-form RNAP, the σ_NCR_ and β’CT interaction in the RPc closes the β’clamp, which likely stimulates the ejection and prevents re-entry of σ_1.1_ due to its steric clash with the β’ clamp (Fig. 5b).

σ_NCR_ contains a highly negatively charged region (acidic loop, residues 167–213) (Supplementary Fig. 8a). Its conformation has not been determined due to its dynamic behavior, but since it is located near σ_2.3_, it seems to prevent nonspecific DNA binding to σ_2.3_ (Supplementary Fig. 8b). We speculate that after RNAP recognizes the UP and −35 elements, loading of the −10 element DNA onto σ domain 2 triggers σ_NCR_ rotation due to charge-charge repulsion. After DNA unwinds around the −10 element, σ_NCR_ returns to its position, as seen in the RPo akin to the apo-form RNAP, and may enhance the electrostatic interaction between σ_2_ and −10 element DNA (Supplementary Fig. 8c). Consistently, deletion of the acidic loop (Δσ_AL_) had a weak destabilizing effect on the *rrnB*P1-RNAP complex, without strong effects on DksA inhibition (Fig. 3 and Table 1).

In addition to the promoter DNA bound on the holoenzyme surface of RPc, a second DNA molecule was accommodated in the RNAP cleft, akin to the downstream DNA of the RPo. We argue that the double-stranded DNA binding in the RNAP cleft is the consequence but not the cause of σ_1.1_ ejection and the σ_1.2_/σ_NCR_ changes. First, the presence of σ_1.1_ at the RNAP cleft in the apo-holoenzyme prevents any DNA binding at this cleft; therefore, σ_1.1_ ejection has to be completed prior to binding of DNA and it requires disruption of the βGL and σ contacts coupled to the σ_NCR_ rotation. Second, none of the available promoter complex cryo-EM structures containing double-stranded DNA at the RNAP cleft show the σ_NCR_ rotation^14,40^ suggesting that the downstream DNA binding by itself is unlikely to trigger the conformational change in σ_1.2_/σ_NCR_. Thus, the RPc structure illustrates how the downstream DNA cleft in RNAP becomes prepared for binding of the downstream DNA duplex during open complex formation.

DksA/ppGpp binding to RNAP also partially opens the DNA loading gate by moving the βlobe/Si1 away from σ_1.1_/σ_1.2_, but it is not strictly coupled to the σ_1.1_ ejection from the RNAP cleft (R-DksA, Fig. 5a). Similarly, the structures of the RNAP–TraR complex and several RNAP–DNA complex intermediates prepared in the presence of TraR also showed the opening of the DNA loading gate by shifting the βlobe/Si1 position but did not show σ_1.2_/σ_NCR_ rotation. Furthermore, σ_1.1_ was not ejected from the RNAP cleft at the stage of RPc formation (Supplementary Fig. 9a)^14,38^.

To understand the role of σ_1.1_ in rRNA transcription, we characterized an RNAP derivative lacking σ_1.1_ (Δσ_1.1_-RNAP) in terms of its *rrnB*P1 transcription activity and sensitivity to DksA. Compared to the wild-type (WT) RNAP, Δσ_1.1_-RNAP has an increased *rrnB*P1 complex stability, both in the absence of DksA (increase in t_1/2_ from 34 s to 115 s) and in its presence (increase in t_1/2_ from «10 s to 20 s) (Fig. 3a, b), and decreased sensitivity to DksA (Table 1). At the same time, the Δσ_1.1_ deletion (and, similarly, Δσ_AL_) does not change the transcription start site in *rrnB*P1 suggesting that σ_1.1_ does not affect DNA scrunching during transcription initiation (Fig. 3c). Overall, the results indicate that σ_1.1_ plays an important role in the destabilization of rRNA promoter complexes and their regulation by DksA/ppGpp.

## Discussion

**Fig. 6:**
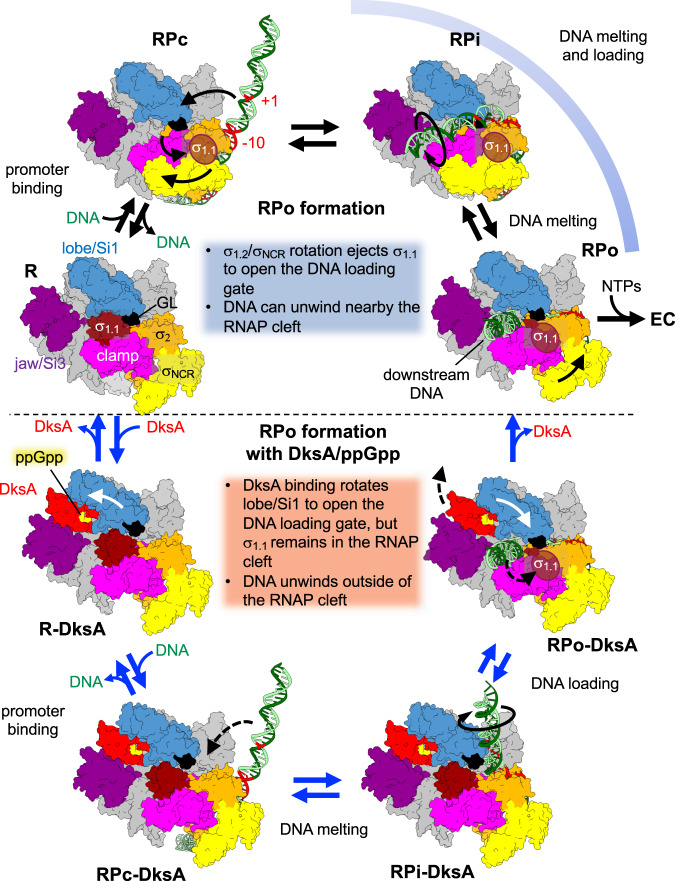
Alternative pathways for open promoter complex formation. Two distinct pathways are shown for open promoter complex formation without (top, blue caption) and with DksA/ppGpp (bottom, red caption). The RNAP holoenzyme (clamp, pink; σ_NCR_, yellow; σ_1.1_, brown; σ_2_, orange; βlobe/Si1, light blue; jaw/Si3, purple; rest of RNAP, gray), promoter DNA (tDNA, dark green; ntDNA, light green), DksA (red), and ppGpp (yellow) are shown. Only RPi is a hypothetical intermediate, but others (RPc, RPo, R-DksA, RPc-DksA, RPi-DksA, and RPo-DksA) represent the structures determined in this and previous studies. EC elongation complex.

Mechanism of rRNA-specific transcription inhibition by DksA/ppGpp. Structural and biochemical studies of bacterial RNAP transcription suggest that the order of DNA loading around the TSS and DNA opening may be interchangeable during promoter recognition (i.e., DNA melts first outside RNAP (melt-load) or DNA melts after loading inside the RNAP cleft (load-melt)) depending on σ factors, promoters, transcription factors and conditions^15,33,41,42^. By combining structural and biochemical data from this and previous studies, we propose two pathways of RPo formation (Fig. 6 and Supplementary Movie 5). We hypothesize that RNAP may use alternative mechanisms of RPo formation with *rrnB*P1 and possibly other promoters, requiring the opening of the DNA loading gate (disrupting the βGL contact to σ), σ_1.1_ ejection from the DNA binding channel, and unwinding of the −10 element plus discriminator DNA, depending on the absence or presence of DksA/ppGpp.

Without DksA/ppGpp (top, RPo formation), free RNAP (R) binds promoter DNA (RPc), which opens the DNA loading gate by ejecting σ_1.1_ from the RNAP cleft, making RNAP competent for melting and loading discriminator DNA (RPi) into the RNAP cleft, which results in efficient RPo formation. The scrunched open complex (RPo) releases RNAP from the rRNA promoter rapidly to proceed with RNA synthesis (EC).

In the presence of DksA/ppGpp (bottom, RPo formation with DksA/ppGpp), DksA/ppGpp binding to RNAP rotates the βlobe/Si1 to DksA, which partially opens the DNA loading gate by disrupting the interaction between GL and σ (R-DksA). However, σ_1.1_ ejection is uncoupled from RPc formation (RPc-DksA), and σ_1.1_ can remain inside the RNAP cleft until the late stages of the open complex formation (RPi-DksA). This pathway favors the melt-load model for RPo formation (RPo-DksA), in which DNA is accommodated above the βlobe domain and unwinds outside the RNAP cleft (Supplementary Fig. 9b) followed by single-stranded tDNA entry into the active site of RNAP^14^. DNA unwinding outside the RNAP cleft is unfavorable in the DksA/ppGpp-free RNAP due to a steric clash of the discriminator DNA with the βlobe. The progression of DNA unwinding from the −10 element to the TSS is energetically less favorable for DksA/ppGpp-sensitive promoters (e.g., *rrnB*P1 and *rpsT*P2) containing the G + C-rich discriminator than for less DksA/ppGpp-sensitive promoters (e.g., T7A1 and RNA1) containing an A + T rich discriminator (Supplementary Fig. 10). *E. coli* promoters that are sensitive to DksA/ppGpp contain G + C-rich discriminators^43^. Replacing the A + T-rich discriminator of the *uspA* promoter, which is positively regulated by DksA/ppGpp, with the one from the *rrnB*P1 promoter makes the *uspA* hybrid promoter sensitive to DksA/ppGpp^44^, indicating that discriminator sequences play an important role in responding to DksA/ppGpp. Although DksA could inhibit transcription regardless of the promoter bound to RNAP, by inhibiting NTP entry and folding trigger helix, stable promoter complex formation decreases DksA binding to RNAP, thus relieving the inhibition^11,36^. The completion of discriminator DNA loading into the RNAP cleft can likely occur not only in DksA/ppGpp-insensitive promoters but also in a fraction of the rRNA promoter complexes to maintain a basal level of rRNA expression under stress growth conditions. This likely pushes the βlobe/Si1 away from the CT-helix of DksA (RPo-DksA), allowing rapid dissociation of DksA from the RNAP secondary channel (RPo) followed by the initiation of transcription (EC).

From this study, we proposed two alternative pathways for opening the gate of the DNA binding channel of RNAP depending on the absence or presence of DksA/ppGpp (Fig. 6 and Supplementary Movie 5) and shed light on the functions of σ_1.1_, σ_1.2,_ σ_NCR_, and βlobe/Si1 domains to explain how DksA/ppGpp specifically inhibits rRNA transcription. Intriguingly, DksA/ppGpp is able to activate transcription at some σ^70^-promoters^45^ and promoters recognized by alternative σ factors, including σ^S^^46^ and σ^E^^47^. Neither σ^S^ nor σ^E^ contains σ_1.1_ or σ_NCR_, and the σ^S^ and σ^E^ holoenzymes use the βGL to close the DNA loading gate^48,49^. DNA binding to the σ domain 2 of σ^S^ or σ^E^ cannot facilitate the opening of the DNA loading gate, as described in the case of the σ^70^ holoenzyme (Fig. 6, top). However, DksA/ppGpp binding followed by the movement of the βlobe/Si1 domain could still open the DNA loading gate of these RNAP holoenzymes as described above (Fig. 6, bottom), possibly explaining the stimulatory effects of DksA/ppGpp on transcription from some σ^S^- and σ^E^-dependent promoters. Further structural analyses of the σ^70^, σ^S^, and σ^E^ RNAP promoter complexes with DksA/ppGpp together with their biochemical characterization will be needed to complete our understanding of DksA/ppGpp-dependent transcription regulation.

## Methods

### Preparation of *rrnB*P1 DNA

The *rrnB*P1 promoter DNA was synthesized (IDT) according to the native *rrnB*P1 sequence and annealed in a 40 μL reaction mixture containing 10 mM Tris-HCl (pH 8.0), 50 mM NaCl, and 1 mM EDTA to a final concentration of 0.5 mM. The solution was heated at 95 °C for 10 min, and then the temperature was gradually decreased to 22 °C. The sequence of the nontemplate strand is 5′-CAGAAAATTATTTTAAATTTCCTCTTGTCAGGCCGGAATAACTCCCTATAATGCGCCACCACTGACACGGACTCTACGAG-3′. The transcription start site is underlined, and the template sequence is 5′-CTCGTAGAGTCCGTGTCAGTGGTGGCGCATTATAGGGAGTTATTCCGGCCTGACAAGAGGAAATTTAAAATAATTTTCTG-3′.

### Cryo-EM sample preparation

*E. coli* σ^70^ RNAP holoenzyme and DksA were purified, as described previously^11,50^. To prepare the RNAP and *rrnB*P1 promoter complex, *E. coli* σ^70^ RNAP (20 μM) and *rrnB*P1 promoter DNA (40 μM) were preincubated for 5 min at 37 °C in buffer (10 mM HEPES, pH 8.0, 50 mM NaCl, 0.1 mM EDTA, 5 mM DTT and 5 mM MgCl_2_). This sample was used for determining the cryo-EM structure of RPo (Supplementary Fig. 3). To stabilize the RPo, NTPs (ATP and a nonhydrolyzable CMPCPP (cytidine-5′-[(α,β)-methyleno]triphosphate, Jena Bioscience) (2 mM each) were added to the RNAP-*rrnB*P1 promoter complex; however, this condition formed the RPc and RPtic (Supplementary Fig. 2) as described in “Results”.

To prepare RP-DksA/ppGpp (Supplementary Fig. 6), *E. coli* σ^70^ RNAP (20 μM) was preincubated with a fivefold molar excess of DksA (100 μM) and ppGpp (2 mM) for 5 min at 37 °C in the buffer (10 mM HEPES, pH 8.0, 50 mM NaCl, 0.1 mM EDTA, 5 mM DTT, and 5 mM MgCl_2_). The *rrnB*P1 promoter DNA (40 μM) was added to the reaction and further incubated for 5 mins at 37 °C. Before freezing the grids, 8 mM CHAPSO (Hampton research) was added to the reaction. A 3.5 μL sample was applied to a glow-discharged C-Flat Holey Carbon grid (Cu 2/1, 400 mesh), blotted and plunge-frozen in liquid ethane using a Vitrobot Mark IV (FEI, USA) with 95% humidity at 4 °C.

### Cryo-EM data acquisition

The grid was imaged using a 300 keV Titan Krios (Thermo Fisher) microscope equipped with a K3 direct electron detector (Gatan) and controlled by the Latitude S (Gatan, Inc.) software at the National Cancer Institute’s Cryo-EM Facility at Frederick. The defocus range was −1.0 to −3.0 µm, and the magnification was ×81,000 in electron counting mode (pixel size = 1.08 Ǻ/pixel). Forty frames per movie were collected with a dose of 1.125 e^−^/Å^2^/frame, giving a total dose of 45 e^−^/Å^2^.

### Cryo-EM data processing

The RNAP-*rrnB*P1 complex with ATP/CMPCPP data was processed using Relion3.0.8^51^. A total of 8315 movies were collected, aligned, and dose weighted using MotionCor2^52^. CTF fitting was performed with Gctf^53^. Initially, ~1000 particles were manually picked to generate particle templates followed by automated picking, resulting in a total of 1,449,010 particles subjected to 2D classification. From the 2D classes, 1,442,810 particles were chosen for the 3D classification to four classes. Poorly populated classes were removed, resulting in datasets of 541,257 (37%) particles for the first class (RPc) and 464,512 (32%) particles for the second class. The first class was further 3D classified without alignments twice to further clean the data, resulting in datasets of 67,187 particles. The particles were refined and postprocessed to generate the density map at 4.14 Å resolution. The resolution of the density map of the second class was 3.53 Å.

The RNAP-*rrnB*P1 complex data were processed using Relion3.0.8. A total of 4748 movies were collected, aligned and dose weighted using MotionCor2. CTF fitting was performed with Gctf. Approximately 1000 particles were manually picked to generate particle templates followed by automated picking, resulting in a total of 563,500 particles. Particles were 2D classified, and 561,753 particles were chosen for the 3D classification. Of the four 3D classes, class 1 (RPo) was the most populated class (349,752 particles, 62%) and was autorefined. The map was postprocessed to give a structure of RPo at 3.53 Å.

The RP-DksA/ppGpp complex data were processed using cryoSPARC V2.9.0^54^. A total of 4,926 movies were collected, and the movies were aligned, and dose weighted using Patch-motion correction. CTF fitting was performed with Patch-CTF estimation. Initially, ~1000 particles were manually picked to generate particle templates followed by automated picking, resulting in a total of 418,049 particles subjected to 2D classification. After two rounds of 2D classification to remove junk particles, 361,048 particles were used to generate two ab initio models. Junk particles were removed, resulting in a dataset of 275,629 particles chosen for the 3D classification (heterogenous refinement). Poorly populated classes were removed, resulting in a dataset of 49,995 particles to generate the density map at 3.62 Å resolution for the first class (RP1-DksA/ppGpp) and a dataset of 79,275 particles to generate the density map at 3.58 Å resolution for the second class (RP2-DksA/ppGpp). The particles were 3D autorefined without the mask and postprocessed (homogenous refinement).

### Structure refinement and analysis

To refine the closed and open complex structures, the *E. coli* RNAP holoenzyme crystal structure (PDB: 4YG2) was manually fit into the cryo-EM density map using Chimera^55^ and real-space refined using Phenix^56^. In the real-space refinement, the domains of RNAP were rigid-body refined and then subsequently refined with secondary structure, Ramachandran, rotamer, and reference model restraints. To refine the structures of RP1-DksA/ppGpp and RP2-DksA/ppGpp, *E. coli* RNAP and DksA/ppGpp complex crystal structures (PDB: 5VSW) were manually fit into the cryo-EM density map using Chimera. DNA was manually built by using Coot^57^. The structure was refined by the same method as the closed and open complex structures. To superpose the structures of RNAP, we used the αNTDs and the catalytic domains (DPBB domains from the β and β’ subunits) of RNAP as references. Figures were prepared by ChimeraX^58^ and Pymol.

### Preparation of RNAP and transcription factors for in vitro transcription

Mutant variants of RNAP, σ^70^, and DksA were obtained by site-directed mutagenesis. The sequences of all primers used in this study are shown in Supplementary Table 2. The D256A substitution in the β′ subunit was obtained in pVS10 encoding all RNAP subunits, with the *rpoC* gene containing a C-terminal His_6_-tag^59^. The σ^70^ and DksA variants containing an N-terminal His_6_-tag were cloned into pET28. To obtain σΔ_1.1_, the 5′-terminal part of the *rpoD* gene encoding residues 2–94 was deleted. To obtain σΔ_AL_, codons 168-212 were replaced with three glycine codons. All proteins were expressed in *E. coli* BL21(DE3). The wild-type and mutant core RNAPs were purified using Polymin P precipitation followed by heparin (HiTrap Heparin column), Ni-affinity (HisTrap HP column), and anion exchange (MonoQ column) chromatography steps (all columns from GE Healthcare)^59^. The wild-type and mutant σ^70^ factors were purified from inclusion bodies with subsequent renaturation and Ni-affinity chromatography^34^. The σΔ_1.1_ protein was subjected to thrombin protease (GE Healthcare) treatment in PBS buffer (10 h of incubation at 4 °C with ten units of protease per mg of protein), followed by incubation with Ni-NTA agarose (GE Healthcare) to remove the His-tag and His-tagged thrombin. To purify DksA, bacterial pellet from 0.5 liters of cell culture was resuspended in 25 ml of lysis buffer (50 mM Tris-HCl, pH 7.9, 250 mM NaCl, 10 mM EDTA, 0.5 mM phenylmethylsulfonyl fluoride, 1 mM 2-mercaptoethanol, 0.1 mM ZnCl_2_) and lysed using a French press. The supernatant obtained after centrifugation was loaded onto a 5-ml HiTrap chelating column (GE Healthcare) charged with Ni^2+^ and equilibrated with loading buffer (10 mM Tris-HCl, pH 7.9, 500 mM NaCl, 0.5 mM 2-mercaptoethanol, 0.1 mM ZnCl_2_). The column was washed with the same buffer containing 60 mM imidazole, and DksA was eluted with buffer containing 300 mM imidazole and dialyzed overnight against 50 mM Tris-HCl, 300 mM NaCl, 1 mM DTT, and 0.1 mM ZnCl_2_. Glycerol was added to 50%, and aliquots were stored at −70 °C.

### Transcription in vitro

Analysis of transcription in vitro was performed using a supercoiled pTZ19 template containing *rrnB*P1 cloned 88 nt upstream of the *his* terminator; the second transcript monitored in the assays was 110 nt RNAI encoded by the *ori* region of the plasmid^30^. For measurements of promoter complex stabilities, promoter complexes were prepared by mixing core RNAP (100 nM final concentration) with wild-type or mutant σ^70^ factors (250 nM) in transcription buffer (40 mM Tris-HCl, pH 7.9, 10 mM MgCl_2_, 40 mM KCl) and supercoiled plasmid DNA (10 nM), followed by incubation for 7 min at 37 °C. DksA and ppGpp were added at 2 μM and 200 μM, respectively, when indicated. An upstream fork-junction competitor DNA was added (template strand 5′-ACGAGCCGGAAGCAT-3′, nontemplate strand 5′-ATGCTTCCGGCTCGTATAATGTGTGGAA-3′; the −10 sequence is underlined) to 2 μM, and the samples were incubated at 37 °C for the indicated time intervals. NTP substrates were added to final concentrations of 200 μM ATP, CTP, GTP, and 10 μM UTP, with the addition of α-[^32^P]-UTP together with rifapentin (5 μg/ml) to prevent re-initiation. The reactions were stopped after 5 min with 8 M urea and 20 mM EDTA, and RNA products were separated by 15% denaturing PAGE, followed by phosphorimaging using a Typhoon 9500 scanner (GE Healthcare). To calculate the observed half-life times for promoter complex dissociation (*t*_1/2_), the data were fitted to the one-exponential equation *A* = *A*_0_×exp(–t×*k*_obs_), where A is the RNAP activity at a given time point after competitor addition, A_0_ is the activity measured in the absence of the competitor, *k*_obs_ is the observed rate constant, and *t*_1/2_ = ln2/*k*_obs_.

For measurements of apparent DksA affinities, promoter complexes were prepared in the same way with 50 nM core RNAP, 250 nM σ^70^ (250 nM), and 2 nM supercoiled plasmid DNA in transcription buffer containing 100 μg/ml BSA for 7 min at 37 °C, followed by the addition of DksA (from 10 nM to 10 μM), either in the absence or in the presence of ppGpp (100 μM). Transcription was performed for 15 min at 37 °C with 200 μM ATP, CTP, GTP, and 10 μM UTP (plus α-[^32^P]-UTP), and RNA products were analyzed as described above. The apparent dissociation constant values (*K*_d,app_) were calculated from the hyperbolic equation: A = A_max_ × (1 – [DksA]/(*K*_d,app_ + [DksA])), where A is the observed RNAP activity and A_max_ is the RNAP activity measured in the absence of DksA.

For transcription start site mapping, in vitro transcription was carried out with wild-type and mutant RNAPs (100 nM core enzyme, 500 nM σ^70^) in the transcription buffer for 15 min at 37 °C with the pTZ19*rrnB*P1 plasmid (25 nM) in the presence of 200 μM ATP, GTP, CTP and 20 μM UTP. RNA was ethanol precipitated, dissolved in water, mixed with 1 pmol of 5′-^32^P-labeled primer (corresponding to positions from +30 to +10 of the initially transcribed region in *rrnB*P1), incubated at 65 °C for 2 min, and chilled on ice. The reverse transcription buffer, dNTPs, RiboLock, and Maxima Reverse Transcriptase (Thermo Scientific) were added in accordance to the manufacturer’s instructions, and the mixtures were incubated for 30 min at 50 °C. The samples were mixed with stop buffer (8 M urea, 20 mM EDTA, 2×TBE) and analyzed by 20% PAGE (19:1) together with radiolabeled oligonucleotide markers.

### DNA duplex free energy calculation

DNA duplex free energies were analyzed based on nearest-neighbor thermodynamics^60,61^. Briefly, the Python script was written to read a sequence from a text file, calculate the DNA duplex free energy of dinucleotides, sum these values over an 8-base window and report these sums for the first base of the central nucleotide of the window (e.g., the sum for the first window base 1–8 will be reported for base 4).

### Reporting summary

Further information on research design is available in the Nature Research Reporting Summary linked to this article.

## Supplementary information

Supplementary Information

Peer Review File

Reporting Summary

Description of Additional Supplementary Files

Supplementary Movie 1

Supplementary Movie 2

Supplementary Movie 3

Supplementary Movie 4

Supplementary Movie 5

## Data Availability

The data that supports this study are available from the corresponding author upon reasonable request. The cryo-EM density maps have been deposited in EMDataBank under accession codes EMDB: EMD-21879 (RPc), EMD-21880 (RPo), EMD-21881 (RP1-DksA/ppGpp), and EMD-21883 (RP2-DksA/ppGpp). Atomic coordinates for the reported cryo-EM structures have been deposited with the Protein Data Bank under accession numbers 7KHC (RPc), 7KHB (RPo), 7KHI (RP1-DksA/ppGpp), and 7KHE (RP2-DksA/ppGpp). Source data are provided with this paper.

## Source data

Source Data
