# Supplementary material for: Structural basis of ribosomal RNA transcription regulation
Source: Nat Commun. 2021 Jan 22;12:528. doi: 10.1038/s41467-020-20776-y (PMC7822876; doi:10.1038/s41467-020-20776-y)
Supplement: Supplementary file 1 — Supplementary Information [file 41467_2020_20776_MOESM1_ESM.pdf]

## Supplementary Table 1. Cryo-EM data collection, refinement and validation statistics

| | RPc (EMD-21879) (7KHC) | RPo (EMD-21880) (7KHB) | RP1-DksA/ppGpp (EMD-21881) (7KHI) | RP2-DksA/ppGpp (EMD-21883) (7KHE) |
|---|---|---|---|---|
| Data collection and processing | | | | |
| Magnification | 81,000 | 81,000 | 81,000 | 81,000 |
| Voltage (kV) | 300 | 300 | 300 | 300 |
| Electron exposure ($e^-/Å^2$) | 45 | 45 | 45 | 45 |
| Defocus range (μm) | -1.0 to -2.5 | -1.0 to -2.5 | -1.0 to -2.5 | -1.0 to -2.5 |
| Pixel size (Å) | 1.08 | 1.08 | 1.08 | 1.08 |
| Symmetry imposed | C1 | C1 | C1 | C1 |
| Initial particle images (no.) | 1,442,810 | 561,753 | 275,629 | 275,629 |
| Final particle images (no.) | 67,187 | 349,752 | 49,995 | 79,275 |
| Map resolution (Å)<br>FSC threshold | 4.14<br>0.143 | 3.53<br>0.143 | 3.62<br>0.143 | 3.58<br>0.143 |
| Map resolution range (Å) | 3.7-10.0 | 3.8-11.0 | 3.2-7.8 | 3.1-8.0 |
| | | | | |
| Refinement | | | | |
| Initial model used (PDB code) | 4YG2 | 4YG2 | 5VSW | 5VSW |
| Model resolution (Å)<br>FSC threshold | 4.1<br>0.143 | 3.5<br>0.143 | 3.6<br>0.143 | 3.6<br>0.143 |
| Map sharpening *B* factor ($Å^2$) | -110 | -125 | -75 | -80 |
| *Model composition*<br>Non-hydrogen atoms<br>Protein residues<br>Ligands | <br>33,509<br>3,830<br>Zn:2, Mg:1, 1N7:2, POP:1 | <br>31,608<br>3,688<br>Zn:2, Mg:1, 1N7:2 | <br>32,349<br>3936<br>G4P:2, Zn:3, Mg:1, 1N7:4 | <br>32,527<br>3850<br>G4P:2, Zn:3, Mg:1, 1N7:4 |
| *B factors ($Å^2$)*<br>Protein<br>Nucleotide<br>Ligand | <br>101.56<br>329.57<br>84.82 | <br>79.45<br>217.76<br>80.86 | <br>107.96<br>264.19<br>104.63 | <br>104.67<br>233.99<br>103.93 |
| *R.m.s. deviations*<br>Bond lengths (Å)<br>Bond angles (°) | <br>0.006<br>0.905 | <br>0.006<br>0.914 | <br>0.004<br>0.894 | <br>0.005<br>0.905 |
| *Validation*<br>MolProbity score<br>Clash score<br>Rotamer outliers (%) | <br>2.30<br>17.96<br>0.00 | <br>2.27<br>18.36<br>0.06 | <br>2.22<br>14.40<br>0.00 | <br>2.27<br>16.70<br>0.00 |
| *Ramachandran plot*<br>Favored (%)<br>Allowed (%)<br>Disallowed (%) | <br>90.00<br>9.29<br>0.71 | <br>91.47<br>7.96<br>0.57 | <br>89.92<br>9.14<br>0.94 | <br>90.21<br>9.03<br>0.76 |
| Model vs. Data<br>CC (mask)<br>CC (box)<br>CC (peak)<br>CC (volume)<br>Mean CC for ligands | <br>0.83<br>0.81<br>0.77<br>0.82<br>0.79 | <br>0.84<br>0.81<br>0.79<br>0.83<br>0.76 | <br>0.83<br>0.78<br>0.74<br>0.82<br>0.73 | <br>0.83<br>0.79<br>0.74<br>0.82<br>0.74 |

**Supplementary Table 2. Oligonucleotide primers**

| Name | Sequence (5' to 3') | Comment |
|---|---|---|
| EC_D256A_d | CTGGTTCCGCTGGCTGGTGGTCGTTTCGCGAC | β´_D256A substitution. PCR 1: EC_D256A_d + EC_BsmI_r on the pVS10 template. PCR 2: PCR1 + EB_ G1260D_d on the pVS10 template. Cloning BsmI/SbfI in pVS10. |
| EC_BsmI_r | GGTGTACATGATCTGGTCCGC | |
| EB_ G1260D_d | CAGCAGCCGCTGGATGGTAAGGCACAGTTC | |
| ED_d168-212_d | ACCGGCTTTGTTGACGGTGGCGGTGACCCGGAACTGGCTCGCG | $\sigma^{70}$_168-212_3Gly deletion. PCR1: ED_d168-212_d+ ED_MfeI_r on pET28*rpoD*. PCR2: ED_d168-212_r + T7_d on pET28*rpoD*. PCR3: T7_d + ED_MfeI_r on the PCR1 + PCR2 template. Cloning XbaI/MfeI in pET28*rpoD*. |
| ED_d168-212_r | CCACCGTCAACAAAGCCGGTGATCAGATCGG | |
| T7_d | TAATACGACTCACTATAGG | $\sigma^{70}$ mutagenesis using XbaI site downstream. |
| ED_MfeI_r | TCATCGCAATTGCCGCGTTGAACCAG | $\sigma^{70}$ mutagenesis reverse primer with MfeI site. |
| ED_del2-94_d | GCGGCAGCCATATGACGACTGACCCGGTACGCATG | $\sigma^{70}$ region 1.1 deletion. PCR1: ED_del2-94_d + ED_MfeI_r on pET28*rpoD*. PCR2: PCR1 + T7_d on pET28*rpoD*. Cloning XbaI/MfeI in pET28*rpoD*. |
| DksA_D137A_r | GCGTTTTGCAGGCGATGCACAGATC | DksA D137A substitution. Circle PCR on pET28*dksA*, phosphorylation with subsequent ligation. |
| DksA140_d | TGGCTGAAATTCGCGAAAAACAG | |

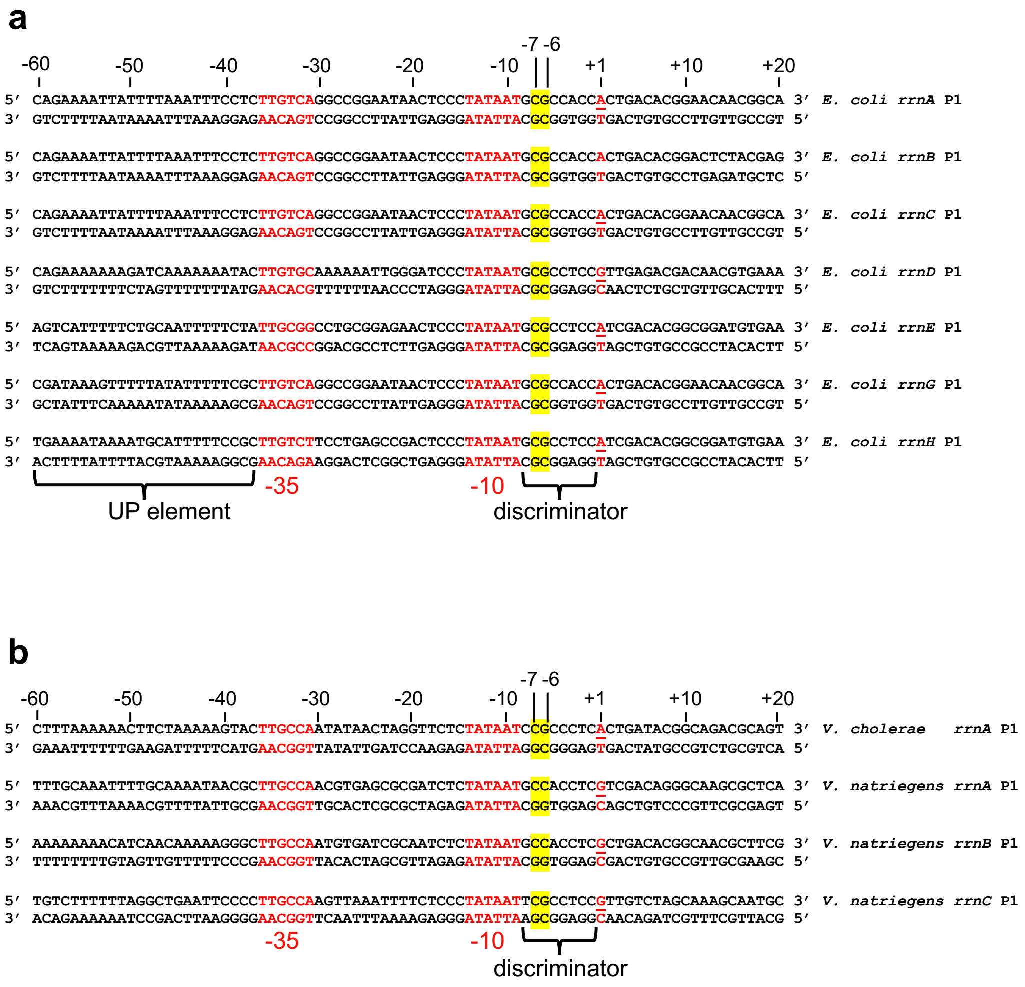


**Supplementary Figure 1. Sequences of rRNA promoters.**

**a**) Sequences of the *E. coli rrnA*P1 – *rrnH*P1 promoter DNAs[1,2].

**b**) Representative rRNA promoter sequences from *Vibrio cholerae* and *Vibrio natriegens* (γ-proteobacteria)[3]. The -35 element, -10 element and transcription start site (TSS, +1) are indicated in red, and the -7 and -6 bases are highlighted in yellow.

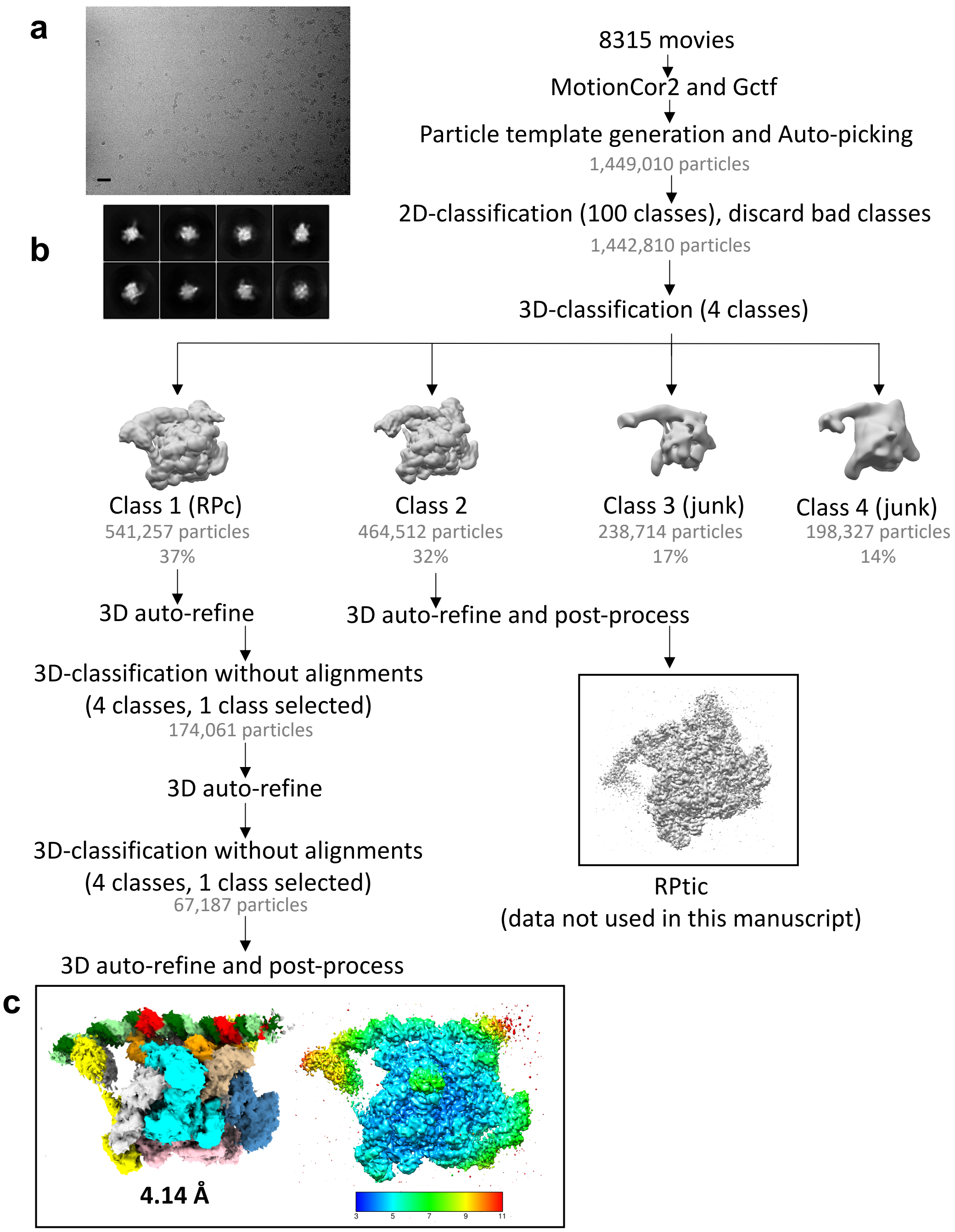


**Supplementary Figure 2. Cryo-EM processing pipeline for the RNAP-*rrnB*P1 complexes (RPc and RPtic) obtained in the presence of iNTPs.**

**a**) A representative micrograph used for data processing. Scale bar is 50 nm.

**b**) Selected classes from the 2D classification.

**c**) The cryo-EM density map for RPc. The left view colored according to Fig. 1b. The right view is the same as on the left but colored by local resolution.

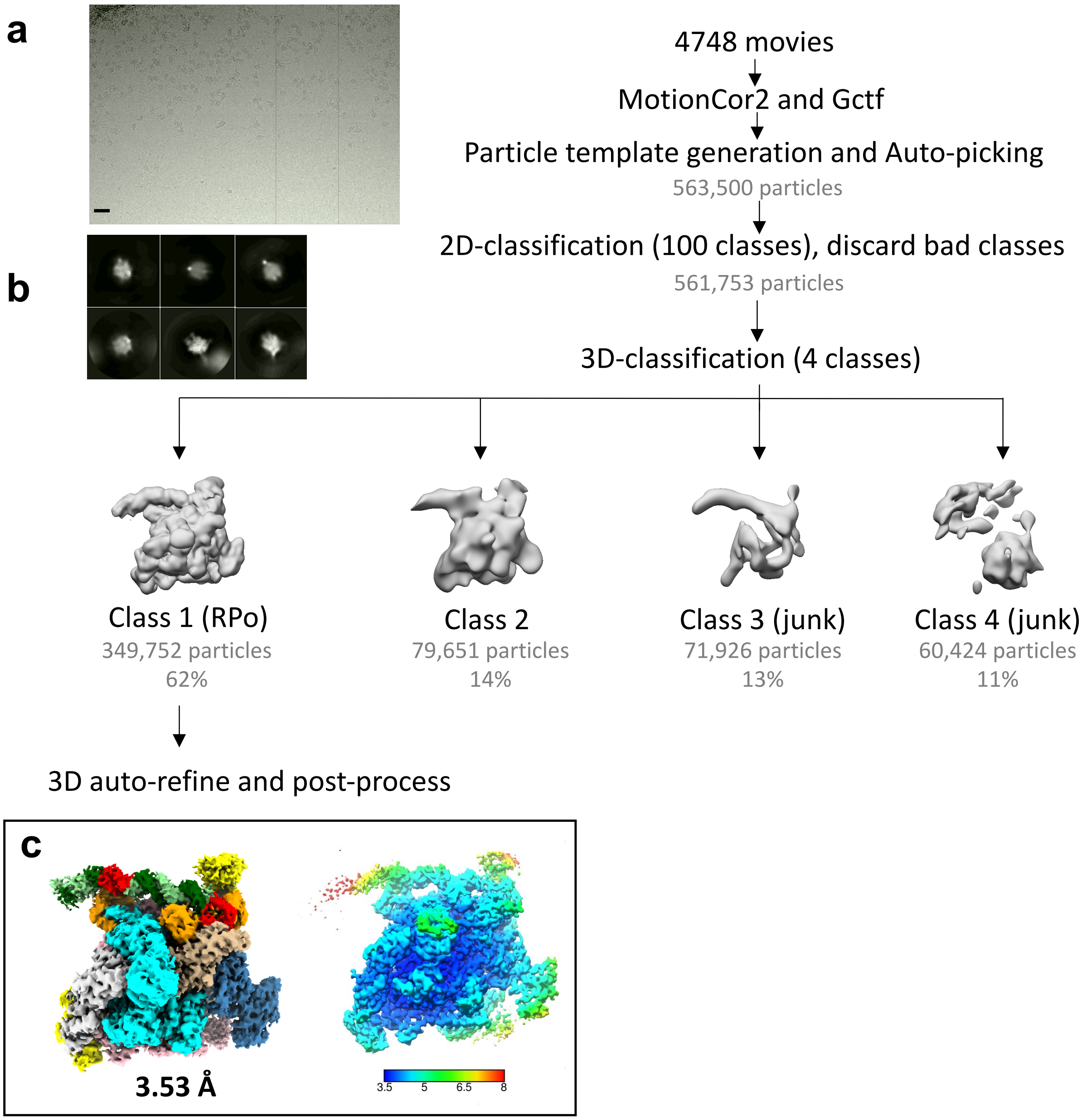


**Supplementary Figure 3. Cryo-EM processing pipeline for the RNAP-*rrnB*P1 RPo complex obtained in the absence of iNTPs.**

**a**) A representative micrograph used for data processing. Scale bar is 50 nm.

**b**) Selected classes from the 2D classification.

**c**) The cryo-EM density map for RPo. The left view is colored according to Fig. 2a; the right view is the same but colored by local resolution.

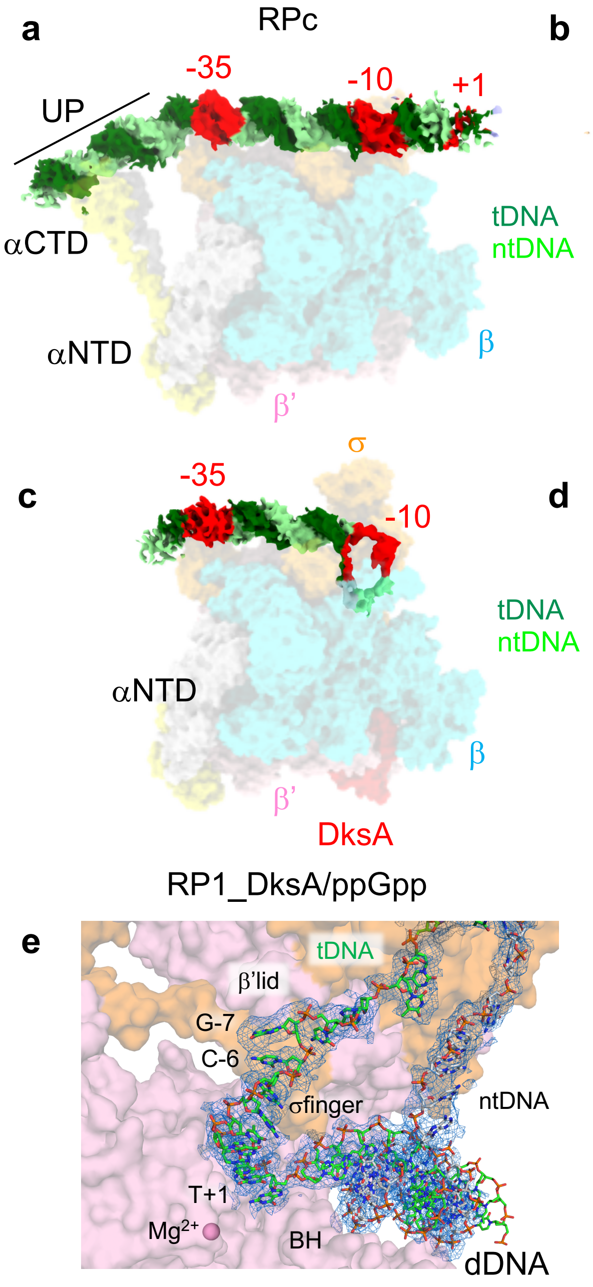


**SFigure 4. Cryo-EM density of promoter DNA from each structure. a-d**) Cryo-EM density maps of the promoter DNA (tDNA, dark green; ntDNA, light green, -35 and -10 elements and +1 transcription start site, red) are shown with transparent RNAP density maps. The cryo-EM density map is colored according to Fig. 4a (α, yellow and white; β, cyan; β', pink; σ, orange; DksA, red). **e)** Cryo-EM density map (blue mesh) of the open DNA bubble in RPo. RNAP (β', pink; σ, orange; β is removed) and DNA are depicted as surface and stick models, respectively. The orientation of this view is the same as in Fig. 2d.

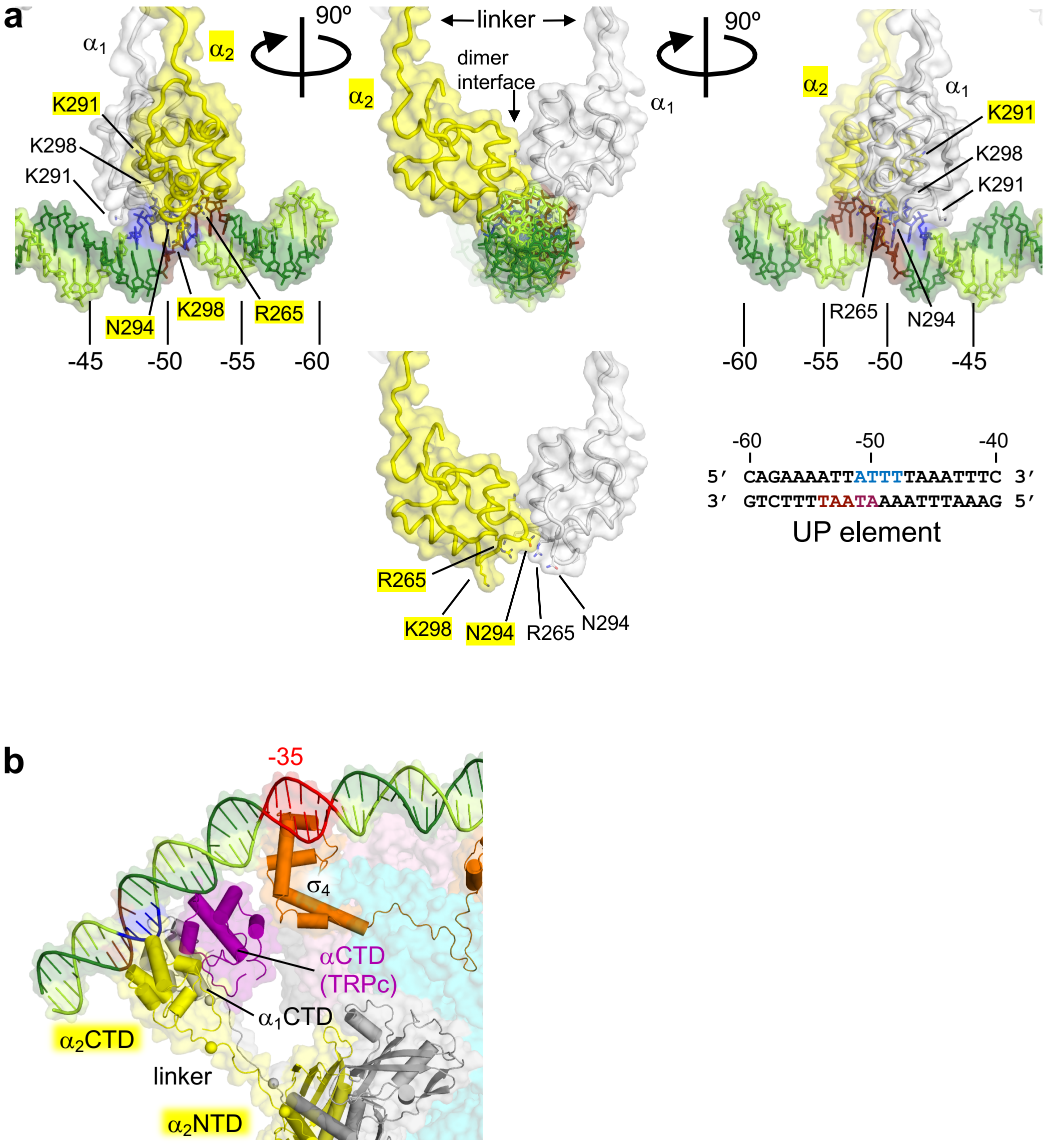


**Supplementary Figure 5. Detailed view of the αCTDs-UP element interaction.**

**a)** Orthogonal views of RNAP contacting the UP element using the head-to-tail αCTD dimer. Th e αCTDs (yellow and white) and DNA around the UP element (dark and light greens) are depicted as cartoon and stick models, respectively. The amino acid residues of the αCTDs and the middle of the UP element (-51 to -48 on ntDNA, blue; -54 to -50 on tDNA, brown) making the interaction are indicated at the bottom. **b**) Comparison of the αCTD and DNA interactions in closed complexes of RNAP with the rRNA promoter *rrnB*P1 (RPc, this study) and the ribosomal protein promoter *rpsT*P2 with TraR (TRPc) (PDB: 6PSQ)[4]. RPc and TRPc are superposed using their αNTDs and catalytic domains (DPBB domains from the β and β' subunits). The figure shows only one of αCTDs (the closest to $\sigma_4$) from TRPc (purple) on the RPc structure. The orientation of this panel and the color scheme are the same as in Fig. 1c. In TRPc, the αCTD monomer binds DNA adjacent to the -35 element, whereas in RPc the αCTD dimer binds in the middle of the UP element (around the -50 bp position).
(Related to Figure 1)

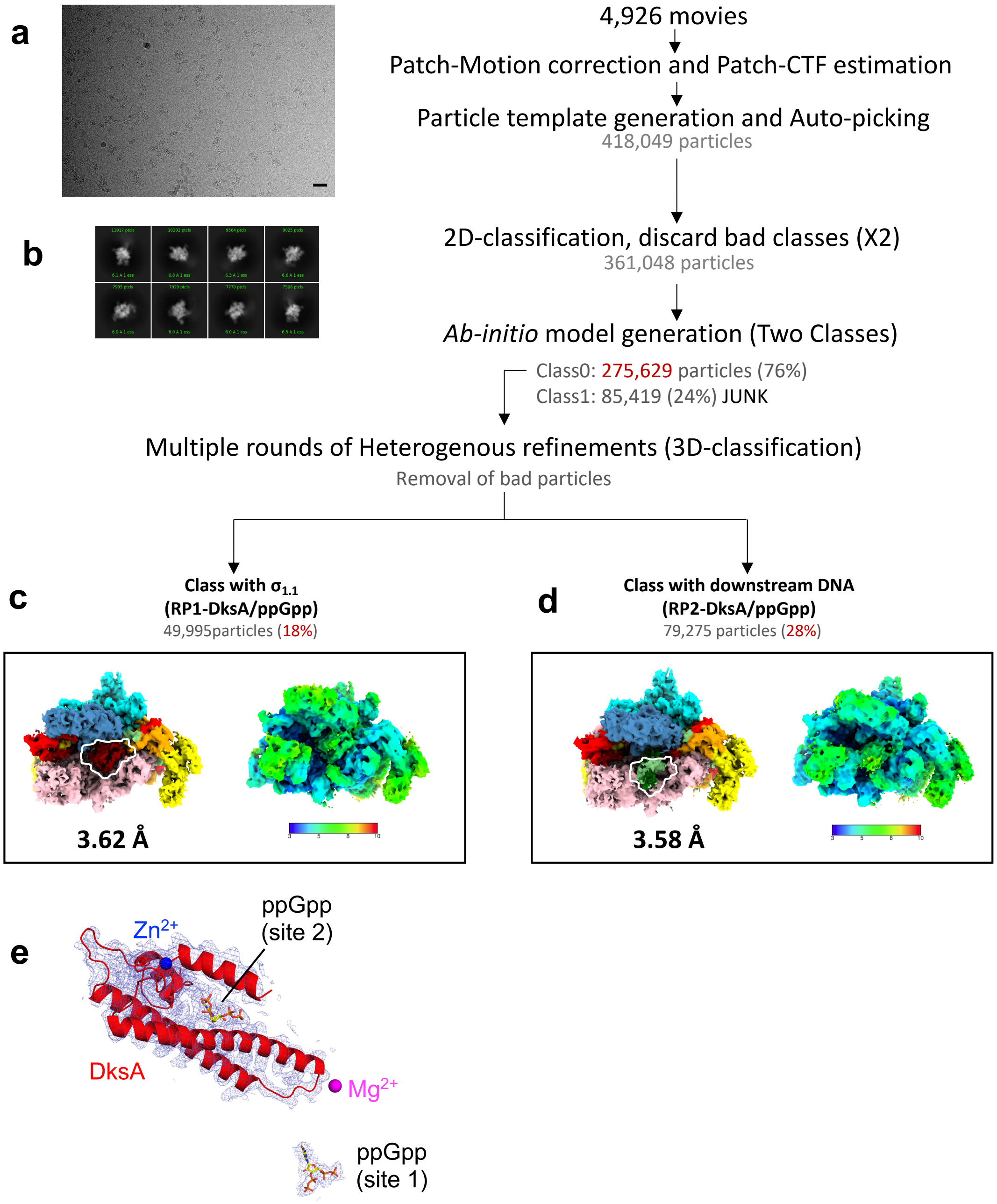


**S Figure 6. Cryo-EM data processing pipeline for the RP-DksA/ppGpp complex. a**) A representative micrograph of the RP-DksA/ppGpp complex used for data processing. Scale bar is 50 nm. **b**) Selected classes from the 2D classification. **c**) RP1-DksA/ppGpp, related to Fig. 4. **d**) RP2-DksA/ppGpp, related to Fig. 4. **e**) Cryo-EM density map (blue mesh) of DksA and ppGpp in the RP2-DksA/ppGpp complex. DksA (red) and ppGpp are depicted as ribbon and stick models, respectively. The position of $Mg^{2+}$ in the RNAP active site is shown as a magenta sphere. The orientation of this view is the same as in Fig. 4B and SFig. 6d.

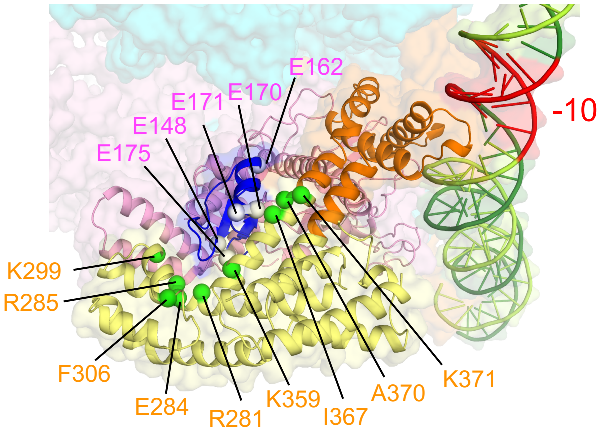


**Supplementary Figure 7. Magnified view of the $\sigma_{NCR}$ and β'clamp-toe (β'CT) interaction in the RPc structure.** The amino acid residues predicted to be involved in the $\sigma_{NCR}$ - β'CT interaction from the previous biochemical study[5] are shown as spheres and labeled.

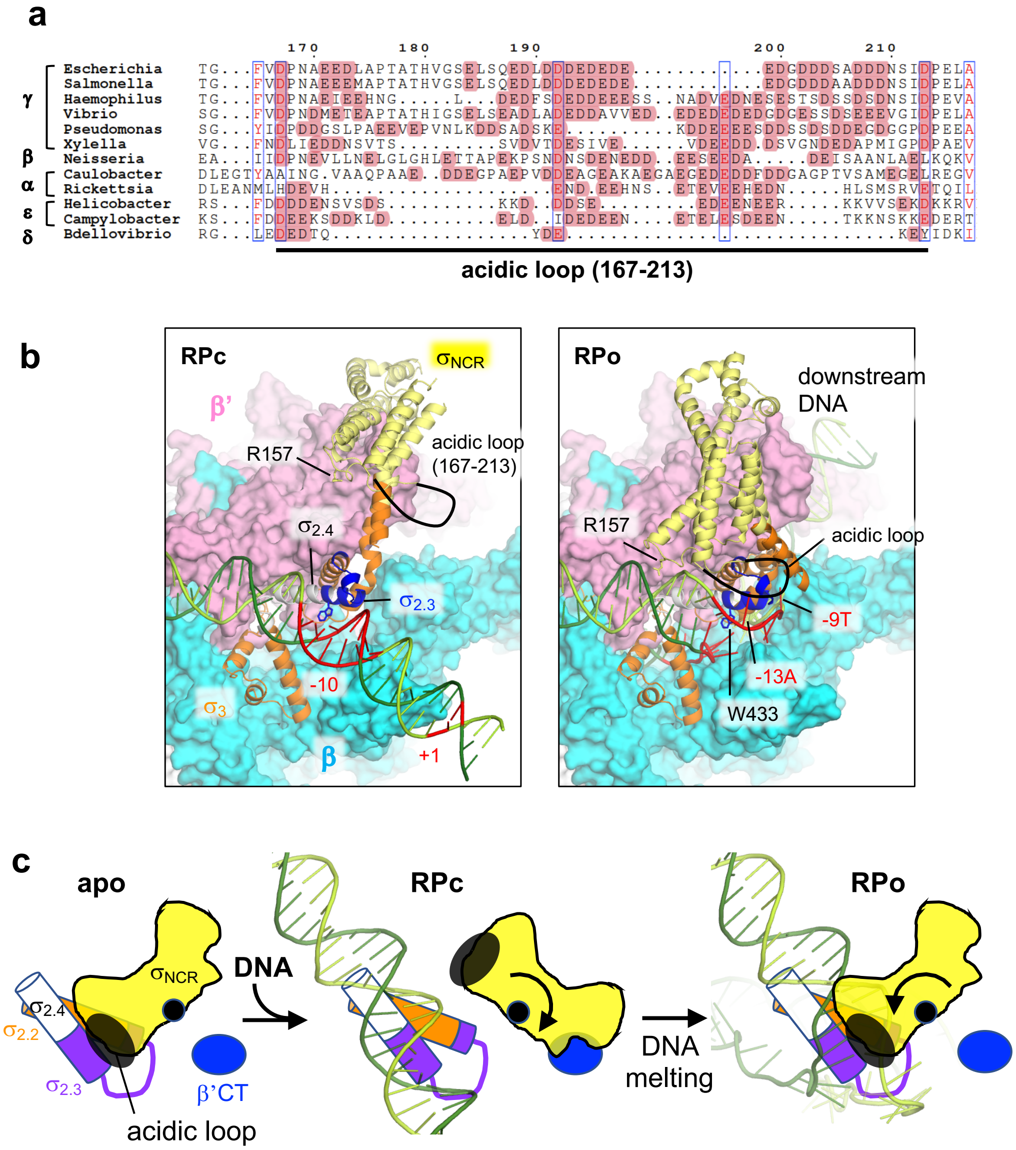


**S Figure 8. Sequence, structure and function of the acidic loop of $\sigma_{NCR}$. a**) Alignment of the $\sigma_{NCR}$ region shows the conservation of the acidic loop in proteobacteria. Acidic residues are highlighted in magenta. **b**) 3D structures showing the σ and -10 element interactions in RPc and RPo. The acidic loop is indicated as a black dashed line. **c**) Proposed role of the acidic loop of $\sigma_{NCR}$ in DNA binding and opening. $\sigma_{NCR}$ (yellow), σ domain 2 ($\sigma_{2.2}$, orange; $\sigma_{2.3}$, purple; $\sigma_{2.4}$, white), β'CT (blue) and DNA (light and dark green) are shown as a cartoon model. In the apo-form R NAP, the acidic loop (black) of $\sigma_{NCR}$ masks $\sigma_{2.3}$, which is unmasked upon the $\sigma_{NCR}$ - β'CT interaction during RPc formation. After DNA binding, the $\sigma_{NCR}$ interaction with DNA may facilitate DNA unwinding.

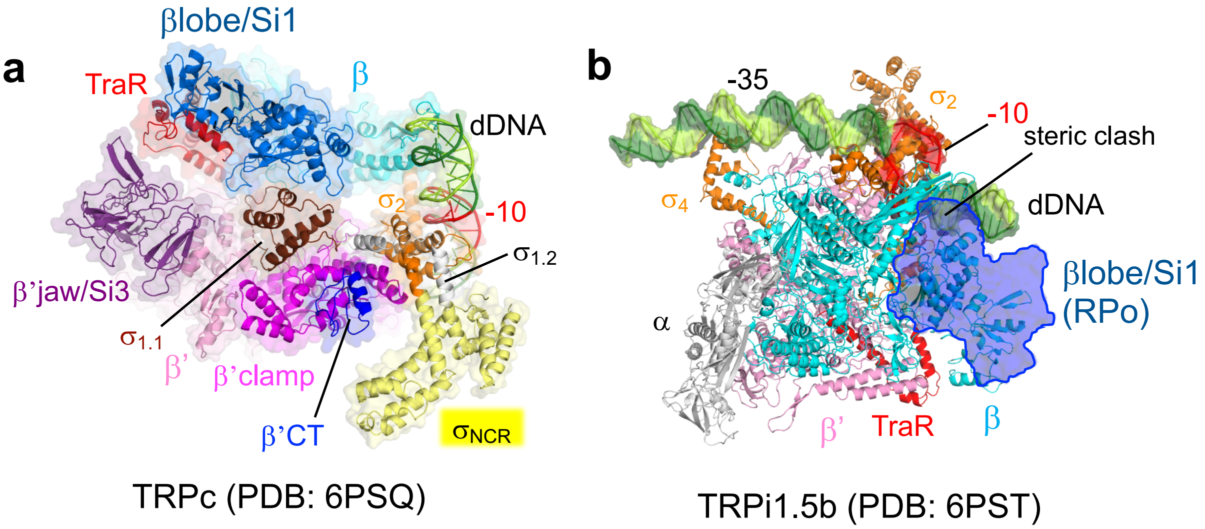


**S Figure 9. Cryo-EM structures of the RNAP – *rpsT*P2 complex with TraR in the closed complex form (TRPc) and in an intermediate form (TRPi1.5b)[4].** RNAP (subunits and domains) and the *rpsT*P2 promoter DNA are shown as cartoon models with transparent surfaces. **a**) The structure of TRPc (PDB: 6PSQ) showed that $\sigma_{1.1}$ and $\sigma_{NCR}$ maintain their positions as in the apo-form of RNAP. **b**) The structure of TRPi1.5b (PDB: 6PST) highlighting accommodation of discriminator DNA above the βlobe/Si1 domain (cyan) in the presence of TraR. The conformation of the βlobe/Si1 domain in the absence of TraR (outlined in blue, RPo) precludes the loading of discriminator DNA above the βlobe/Si1 domain.

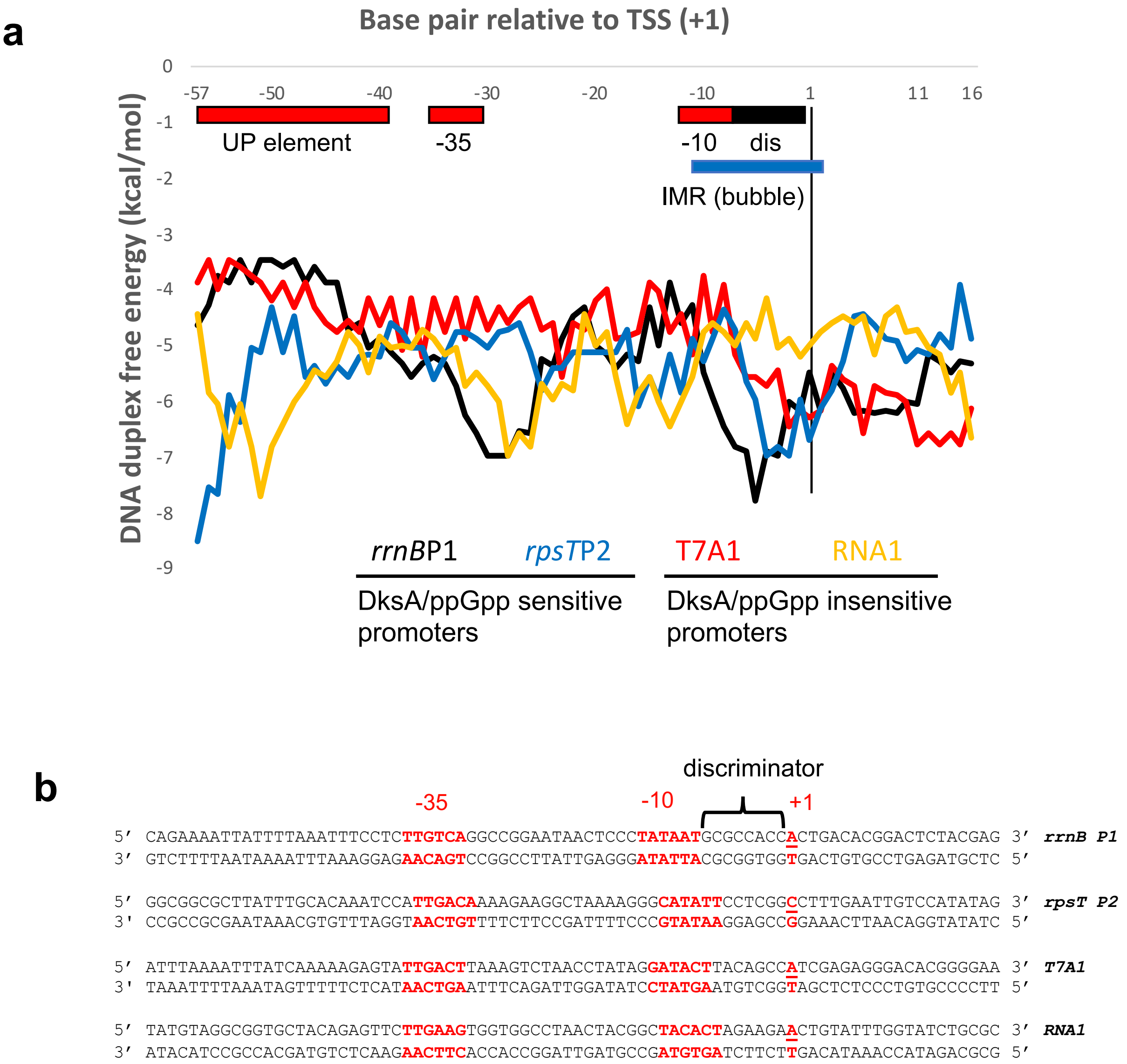


**Supplementary Figure 10. DNA duplex free energy. a**) DNA duplex free energy calculated based on nearest-neighbor thermodynamics[6] for *rrnB*P1 (black), *rpsT*P2 (blue), T7A1 (red) and RNA-I (yellow) promoters. DNA sequences are shown in (**b**). Sequences were aligned at the promoter TSSs. The UP element, -35 and -10 elements, discriminator (dis), and initially melted region (IMR or transcription bubble) are indicated.

## Supplementary REFERENCES


1 Condon, C., Philips, J., Fu, Z. Y., Squires, C. & Squires, C. L. Comparison of the expression of the seven ribosomal RNA operons in Escherichia coli. *EMBO J* **11**, 4175-4185, (1992).

2 Kolmsee, T., Delic, D., Agyenim, T., Calles, C. & Wagner, R. Differential stringent control of Escherichia coli rRNA promoters: effects of ppGpp, DksA and the initiating nucleotides. *Microbiology* **157**, 2871-2879, (2011).

3 Aiyar, S. E., Gaal, T. & Gourse, R. L. rRNA promoter activity in the fast-growing bacterium Vibrio natriegens. *J Bacteriol* **184**, 1349-1358, (2002).

4 Chen, J. *et al.* Stepwise Promoter Melting by Bacterial RNA Polymerase. *Mol Cell*, (2020).

5 Leibman, M. & Hochschild, A. A sigma-core interaction of the RNA polymerase holoenzyme that enhances promoter escape. *EMBO J* **26**, 1579-1590, (2007).

6 SantaLucia, J., Jr. A unified view of polymer, dumbbell, and oligonucleotide DNA nearest-neighbor thermodynamics. *Proc Natl Acad Sci U S A* **95**, 1460-1465, (1998).
