# Supplementary material for: Structural basis of ribosomal RNA transcription regulation
Source: Nat Commun. 2021 Jan 22;12:528. doi: 10.1038/s41467-020-20776-y (PMC7822876; doi:10.1038/s41467-020-20776-y)
Supplement: Supplementary file 4 — Description of Additional Supplementary Files [file 41467_2020_20776_MOESM4_ESM.pdf]

Description of Additional Supplementary Files

Supplementary Movie 1. Cryo-EM density map of the RNAP - *rrnB*P1 closed complex (RPc). Related to Fig. 1b.

Supplementary Movie 2. Cryo-EM density maps of the RNAP - *rrnB*P1 open complex (RPo) and *rrnB*P1 DNA. Related to Fig. 2a.

Supplementary Movie 3. Close-up views of RNAP and discriminator DNA interactions. Related to Figs. 2c and d.

Supplementary Movie 4. Cryo-EM structures of the RNAP - *rrnB*P1 complex with DksA/ppGpp (RP1-DksA/ppGpp and RP2-DksA/ppGpp). Close-up view of the βlobe/Si1 conformational changes upon DksA binding, $\sigma_{1.1}$ ejection and downstream DNA binding. Related to Fig. 4.

Supplementary Movie 5. Alternative pathways for open promoter complex formation. Related to Fig. 6.
