# Supplementary material for: Structural basis of ribosomal RNA transcription regulation
Source: Nat Commun. 2021 Jan 22;12:528. doi: 10.1038/s41467-020-20776-y (PMC7822876; doi:10.1038/s41467-020-20776-y)
Supplement: Supplementary file 2 — Peer Review File [file 41467_2020_20776_MOESM2_ESM.pdf]

Reviewer #1 (Remarks to the Author):

It has been known for many years that the transcription of E. coli rRNA operon is sensitive to growth conditions and DksA/ppGpp serves as the master regulator. However, the underlying mechanism is unknown due to lack of structural information of DksA/ppGpp-engaged transcription initiation complexes. This manuscript reports two serials of cryo-EM structures representing transcription initiation events occurring on rrnBP1 promoter in the presence and absence of DksA/ppGpp. The structures reveal distinct domain movements on the course of RPo formation in the absence and presence of DksA/ppGpp. Two alternative pathways of RPo formation were proposed. Overall, this work represents an important contribution to the understanding of rRNA transcription in bacteria, and I feel it is suited for Nature Communications but requires some revisions:

Major point:
The structure of RPc in the absence of DksA shows another molecular of double-stranded DNA binding to the downstream DNA channel of RNAP in a non-specific manner, which would not occur in physiological conditions in bacterial cells. The presence of the non-specific dsDNA in the channel would inevitably preclude the σR1.1. Therefore, instead of the RPo formation pathway in the absence of DksA/ppGpp proposed by the authors, it is also possible that the non-specific dsDNA first engages the dsDNA-binding channel, ejects the σR1.1, and induces the rotation of the σNCR. Because the σR1.1 ejection is the key point in differentiating the two proposed alternative RPo-formation pathways, the authors should provide a convincing argument that the occupancy of the non-specific dsDNA in the downstream DNA-binding channel is the consequence not the cause of σR1.1 ejection.

Minor points:
1. In Sfigure2, the cryo-EM processing pipeline for RNAP-rrnBP1 complex with ATP/CMPCPP shows roughly 32% single participles were classified into Class 2 and a high-quality map was obtained. Although the author wrote that it was not used in this manuscript, the map suggests that it is a RPo or RPi, which is quite relevant to this study. Could the author briefly describe the structure of class 2?

2. Page 3, line 76 and Page 5, line 133, the author included ATP/CMPCPP for sample preparation, does the map of Class 2 show the presence of the two initiating nucleotides? The previous literature claimed that the high concentration of initiating NTPs is critical for stabilizing rrBP1-RPo [PMID: 9405339], however, it looks that the scrunched rrnBP1-RPo could still be properly formed based on the structure. Does the conclusion still stand?

3. Page 4, the second paragraph, it looks like the UP element-αCTD interaction is different from that in the rpsTP2-RPc structure reported by the Darst's group [PMID: 32160514]. It would be informative to add a supplemental figure for a detailed comparison of the UP element-αCTD interaction in the two structures.

4. Page 4, line 100, although current RPc structure shows no obvious DNA bending upon interaction with the αCTD dimer, however, the interaction of αCTD with a UP element further upstream might induce a significant bending of promoter DNA.

5. Page 5, line 117, does the map support unambiguous modeling of the scrunched template and non-template ssDNA of the transcription bubble? The authors should provide an additional figure with a local map and fitted model to support the scrunched conformation of template strand DNA. Moreover, how is the scrunched nontemplate ssDNA accommodated?

6. Page 5, line 123, there should be a figure citation. The description is also confusing. Is the sR1.1 still present in the RPo complex?

7. Page 6, line 161, the author should provide a local cryo-EM map for the ppGpp and DksA.

8. Fig. 5B, how were the two structures superimposed? Is there any movement of βlobe/Si1? Is there a direct clash between the β′ clamp-toe and σR1.1? If not, how does the rotation of the σNCR induce clamp closure and subsequent σR1.1 ejection?

9. Page 7, line 173, was the interaction of βlobe-SI1/DksA-CT also observed in the previous crystal structure of DksA-RNAP holoenzyme [PMID:29478808]? Were the DksA/ppGpp-induced conformational changes in βlobe/Si1, β′jaw/Si3 and β′clamp also observed in the previous crystal structure of DksA-RNAP holoenzyme?

10. Page 7 and Fig. 3, the model of σR1.2/NCR rotation triggered by σAL/promoter charge-repulsion is attractive. As proposed by the authors in Fig. 6, the ejection of σR1.1 requires rotation of σR1.2/NCR module, therefore, a much larger effect than observed here on RPo formation or RPo stability of σAL deletion would be expected. Have the authors analyzed the σNCR deletion mutant or RNAP-β'CT deletion mutant?

11. Page 13, line 418. There should be two batches of sample preparation for RNAP-rrnBP1 promoter complexes but the text only describes one of them.

12. Page 15, line 453, the defocus range should be "-1.0 to -3.0", also in the STable 1.

Reviewer #2 (Remarks to the Author):

Review:

This very significant manuscript describes 4 moderate resolution (about 4 A) cryoEM structures of complexes of E. coli RNA polymerase and ribosomal (rrnBP1) promoter DNA (-60 to +20).

One complex, formed in the presence of the initiating nucleotide (ATP) and also the -1,-2 dinucleotide (CMPCPP) exhibits scrunched discriminator open strands and a correctly-positioned, open start site for initiation, presumably with bound nucleotides though these apparently are not observable in the structure. Another complex, apparently obtained without nucleotides using the same or similar conditions, is a closed complex which binds duplex DNA to +3. In this closed complex, the two alpha CTD interact with a subregion of the UP element (near -50). This complex, prepared using a 1:2 ratio of RNA polymerase to promoter DNA, has a second DNA molecule bound in the cleft. These structures are a significant addition to those previously available and provide new insights into initiation. But it seems inappropriate to call these structures RPo and RPc, as indicated below.

Two complexes with bound DksA and ppGpp (called RP1-DksA/ppGpp and RP2-DksA-ppGpp) are characterized, revealing aspects of how these factors work.

RP1-DksA/ppGpp shows DksA blocking the secondary channel to prevent the entry of NTP. It also shows a contact between DksA and the beta lobe, proposed to induce the conformational change involving beta lobe/Si1, beta′ jaw/Si3 and beta′ clamp that destabilize the open complex.

RP1-DksA/ppGpp has no detectable downstream DNA density, but the downstream DNA (+3 to +20) is observable in RP2-DksA/ppGpp. The -10 element is open while the discriminator and start site DNA is not observable in in RP2-DksA/ppGpp. The contact between DksA and beta lobe/Si1 is broken, indicating to the authors that the conformation transition between RP1 and RP2 may cause the dissociation of DksA and allow subsequent formation of the initiation complex.

DksA and ppGpp also partially open the DNA loading gate by moving the betalobe/SI1, but not make the ejection of sigma 1.1.

These structures also reveal new information about how sigma region 2 triggers the loading of DNA in the clamp and cleft. From comparison of their -DksA complex structures with that of holoenzyme they deduce that contact of duplex -10 element with sigma region 2 triggers a rotation of the large sigma non-conserved region (NCR) and movement of sigma region 1.2, disrupting the interaction of sigma with the beta gateloop (GL) and allowing the discriminator to enter the RNAP cleft. Unwinding of the -10 element then makes the sigma NCR rotate back to stabilize the open complex.

Comments/Questions:

1) It seems more appropriate to call the open complex RPitc and not RPo, given that it is formed in the presence of NTP and presumably would not be stable enough to observe without bound NTP at +1 and -1,-2.

2) While the text of the manuscript (line 75) seems clear that the closed complex was obtained without nucleotides, the following sentence would then refer to the conditions for preparing the open complex, but is in the section on the closed complex and could be interpreted as meaning a second attempt to make a closed complex. The methods and captions to the SI figures are also ambiguous on this point. In all places, clarification is needed.

Good evidence exists for a series of at least four closed complex intermediates which differ greatly in structure but are very similar in stability on the pathway to open complex formation (see Sreenivasan et al, Biochemistry '20 and refs therein). In all but the initial closed complex, the upstream promoter DNA is found by fluorescence to be highly bent and wrapped (to about -82). The closed complex characterized in the manuscript under review appears different from all closed complexes studied previously, with downstream characteristics like those of a more advanced closed complex and upstream characteristics more like those of the initial closed complex (traditionally called RPc). This may result from destabilization of upstream interactions by truncation at -60 or from perturbations from the second bound DNA and/or the cryoEM sample preparation. It seems important to state the above points explicitly.

The authors do not observe bending and wrapping of the upstream DNA in their open complex, also formed using DNA truncated at -60, whereas AFM, FRET, single molecule force and backbone footprinting all provide strong evidence for upstream wrapping of promoter DNA using a fragment extending to or beyond -100. Hence the absence of upstream wrapping in their closed complex isn't surprising. Since the authors make a point of the lack of upstream bending and wrapping in their closed complex, they should make this comparison for the open complex as well. In doing so it seems worth saying that the lack of upstream wrapping in their open complex could result from destabilization of upstream interactions by truncation at -60 ,or from perturbations of wrapping by binding the initiating NTP, or from the cryoEM sample preparation.

RPc is the designation of the initial closed complex in which the downstream duplex is not strongly bent and makes contacts with RNA polymerase only to about -5, and in which the upstream DNA is also not strongly bent or wrapped. Perhaps the closed complex studied by the authors could be designated RPcc, since it appears quite different than any studied previously.

3) Revisions are needed in lines 45-47 and 148-9. Both talk about facilitation of rrnBP1-promoter escape by reducing the abortive RNA cycle prior to the productive RNA elongation stage, but Shimamoto, Record and others have found for lambdaPr, T7A1 and lacCONS promoters that abortive initiation is performed only by a nonproductive subclass (about 50 %) of initiation complexes which are incapable of promoter escape, and not by productive initiation complexes. For other promoters, Heyduk, Record and others observed correlations between the stability of the open complex and rate of escape and the hybrid length for escape which may be relevant for rrnBP1.

4) Lines 111-2. The authors say sigma region 2.3 fits into the major groove of the duplex DNA in their

closed complex, but that there are no specific contacts between amino acids and DNA bases. Can they be sure of this, given their resolution? Could this lack of specific interaction with the -10 region be caused by the presence of the second bound DNA molecule? Are base specific interactions observed with the -35 region and the UP element, or are these interactions similar to those observed for the -10 element?

5) Figure 3 caption and/or the accompanying text should clarify that the determinations of lifetime of open complexes and stability of DksA interaction are performed with negatively supercoiled DNA. The caption and SI methods should also describe the methods used to determine lifetime/stability and the use of heparin vs. fork junction competitor.

6) Lines 237-9 and other places. In their remarks regarding the -DksA mechanism for open complex formation it would be appropriate to refer to recent structural and solution studies of this mechanism from Darst, Record and other laboratories.

Reviewer #3 (Remarks to the Author):

Regulation of ribosomal RNA (rRNA) transcription during stringent response is critical for bacteria adaptation and survival. The regulation is achieved on the level of transcription initiation on specific rRNA promoters and their interaction with RNA polymerase (RNAP). Moreover, the transcription factor DksA, which directly binds RNAP, drastically effects rRNA promoters activity by sensing the bacterial alarmone ‘ppGpp’. There are two recently published studies revealing series of cryo-EM structures of a DksA homolog, TraR, dependent transcription system (Chen et al, eLife, 2019 and Chen et al, Moll Cell, 2020). However, the latter studies are conducted on different class of bacterial promoters.

In this manuscript, Shin et al reveal important new details specifically of the rRNA transcription regulation based on four cryo-EM structures of rRNA promoter/RNAP complexes. In detail, the authors describe the critical step of the initiation pathway, the formation of so called ‘open complex’ on rRNA promoter, with and without DksA/ppGpp present. By detail comparison of these alternative complexes, the authors propose two plausible pathways of the process. It also allows them to mechanistically describe the role of DksA/ppGpp in rRNA promoter regulation and transcription initiation. These are important advances towards the broad understanding of the rRNA transcription regulation during stringent response.

The added value of the manuscript is also the visualization of the RNAP αCTD subunits interaction with the UP element of DNA promoter. This interaction has been known, but only now characterized structurally.

The manuscript is written in a concise way and well illustrated by five very descriptive movies.

I recommend the manuscript for publication in Nature Communication after the following concerns have been addressed.

1. Line 48: The term ‘optimized and nonoptimized’ growth conditions is not very clear. What kind of optimisation do the authors mean?

2. Line 63: The authors should explain the term ‘stressed RPo’.

3. Line 94-95. The UP element has a conserved sequence. Can the authors explain why is it specifically bound by the αCTDs, while they don’t report any sequence specific contacts? Is there for example any specific shape of the DNA in UP element region, similarly to what is described for the -10 element?

4. Line 137-138: The authors should clarify which promoter-RNAP complex the D256A mutation stabilizes. Do they mean stabilisation of the 'un-scrunched' -3A TSS RPo complex and destabilisation of the regular +1A TSS RPo. Could that be confirmed by the identity of the TSS in the transcription assays in Figure3A?

5. Line 198-199: The authors claim that ''The βGL contacts σ1.1 and σ1.2 to enclose the RNAP cleft in the apo-form RNAP (Fig. 5A) and that 'the same interaction' in RPo stabilises the open complex bubble (Fig. 2B)'. While there is σ1.1 clearly marked in Fig. 5A, it is not in Fig. 2B, and neither 2C. It also seems the σ1.1 is not ordered/visible in neither of the RPo complexes. The authors should clarify role of σ1.1 in open complex bubble stabilisation and clearly mark it in all mentioned figures.

6. Line 218-219: The authors claim the TraR structures did not show σ1.2/σNCR rotation or σ1.1 ejection. In the late stages of TraR mediated RPo formation the σ1.1 is actually ejected (Chen et al., Moll Cell, 2020, Video S3). The authors should clearly specify which state of both systems they are comparing.

7. Line 237-241: The authors describe the pathway of RPo formation without DksA/ppGpp. The first steps include ejection of σ1.1. This is different to what is proposed in Chen et al. (Moll Cell, 2020, Video S4). Can authors explain this discrepancy? Do the authors think the proposed pathway can still be only rRNA promoter dependent?

8. The protein validation statistics in Table S1 reports high clash scores. Generally the clash scores in cryo-EM structures are higher then in X-ray crystallography, but the reported numbers higher then standard. The authors should carefully inspect these and try to correct it. Especially in low resolution regions, where the cryo-em density probably does not define the model so well. The authors should also report cross-correlation of the atomic model with the cryo-EM map.

9. It will probably not change the overall interpretation of the cryo-EM structure analysis, however the authors could try to improve the 3D cryo-EM reconstructions by i) per particle CTF fitting, ii) refinement of optical aberrations and iii) Bayesian polishing and limiting the total fluence to ~26-30 e-/Å2 (Grant, eLife, 2015).

REVIEWER COMMENTS

Reviewer #1 (Remarks to the Author):

It has been known for many years that the transcription of E. coli rRNA operon is sensitive to growth conditions and DksA/ppGpp serves as the master regulator. However, the underlying mechanism is unknown due to lack of structural information of DksA/ppGpp-engaged transcription initiation complexes. This manuscript reports two serials of cryo-EM structures representing transcription initiation events occurring on rrnBP1 promoter in the presence and absence of DksA/ppGpp. The structures reveal distinct domain movements on the course of RPo formation in the absence and presence of DksA/ppGpp. Two alternative pathways of RPo formation were proposed. Overall, this work represents an important contribution to the understanding of rRNA transcription in bacteria, and I feel it is suited for Nature Communications but requires some revisions:

We are grateful for the thorough review and appreciate the referee's recognition of the importance of our work.

Major point:
The structure of RPc in the absence of DksA shows another molecular of double-stranded DNA binding to the downstream DNA channel of RNAP in a non-specific manner, which would not occur in physiological conditions in bacterial cells. The presence of the non-specific dsDNA in the channel would inevitably preclude the σR1.1. Therefore, instead of the RPo formation pathway in the absence of DksA/ppGpp proposed by the authors, it is also possible that the non-specific dsDNA first engages the dsDNA-binding channel, ejects the σR1.1, and induces the rotation of the σNCR. Because the σR1.1 ejection is the key point in differentiating the two proposed alternative RPo-formation pathways, the authors should provide a convincing argument that the occupancy of the non-specific dsDNA in the downstream DNA-binding channel is the consequence not the cause of σR1.1 ejection.

Thank you for your advice. The σNCR rotation and following the σ1.1 ejection from the RNAP cleft is one of important findings from this study. After initial determination of the RPc structure, we also thought about a possibility that the σ1.1 ejection and σNCR rotation are triggered by the DNA binding to the downstream RNAP cleft as this reviewer pointed out. However, after careful analysis of the structures from this and published works, we concluded that these rearrangements are not triggered by the binding of another DNA molecule at the RNAP cleft with the following reasons:
First, σ1.1 is accommodated in the apo-form RNAP holoenzyme to prevent DNA binding to this cleft and, therefore, its ejection should precede the binding of the second DNA molecule and not vice versa.
Second, to compete with σ1.1 (or promoter DNA) the extraneous molecule of DNA should be more stably bound in the downstream cleft than these integrate components of the promoter complex, which is highly unlikely. Indeed, no non-specific DNA binding in the downstream cleft was ever reported in other available structures of RNAP-DNA complexes.
Third, the ejection of σ1.1, which should precede downstream DNA binding, absolutely requires transient opening of the downstream cleft and disruption of the contacts between σNCR and the

βGL, observed in the apo-form holoenzyme RNAP or the RPo structures. The reported RPc structure shows that such opening is coupled with rotation of σNCR, not seen previously.

Fourth, downstream DNA is unlikely to induce the large conformational change in the σNCR in the RPc, and no allosteric pathway that would couple downstream DNA binding to the σNCR rotation could be deduced from the structure. Furthermore, available RPo structures (from this and other studies) containing double-stranded DNA at the downstream RNAP cleft do not show the σNCR rotation as observed in the RPc suggesting that downstream DNA binding cannot by itself induce such changes.

With these facts, we concluded that the RPc formation, particularly the binding of double-stranded DNA around -10 element on the surface of σ domain 2, triggers the σNCR rotation followed by the σ1.1 ejection. Thus, the double-stranded DNA binding in the downstream cleft found in our RPc structure is the consequence but not the cause of σ1.1 ejection. While it is not excluded that the bound downstream DNA molecule may additionally stabilize this particular conformation, its binding is only possible after the aforementioned conformational changes, and the RPc structure illustrates how the RNAP cleft becomes prepared for downstream DNA binding during open complex formation. To provide additional evidence in support of this pathway and to highlight the function of the σNCR rotation during promoter complex formation, we plan to investigate the motions of σNCR and σ1.1 during the RPc and RPo transitions in solution by using single-molecule FRET and report the results in a separate manuscript.

To clarify this point, we modified the text at lanes 258-268:

In addition to the promoter DNA bound on the holoenzyme surface of RPc, the second DNA (double-stranded form) is accommodated in the RNAP cleft, akin to the downstream DNA of the RPo. We concluded that the double-stranded DNA binding in the RNAP cleft is the consequence but not the case of σ1.1 ejection and it does not trigger the σ1.2/σNCR conformational change with following reasons. First, the binding σ1.1 at the RNAP cleft prevents any DNA binding at this cleft, therefore, its ejection has to be completed prior to binding DNA. Second, the RPo structure containing double-stranded DNA at the RNAP cleft did not show the σNCR rotation.

Minor points:

1. In Sfigure2, the cryo-EM processing pipeline for RNAP-rrnBP1 complex with ATP/CMPCPP shows roughly 32% single participles were classified into Class 2 and a high-quality map was obtained. Although the author wrote that it was not used in this manuscript, the map suggests that it is a RPo or RPi, which is quite relevant to this study. Could the author briefly describe the structure of class 2?

In the class 2 complex (now it is called transcript initiation complex, RPtic), de novo synthesized 2-mer RNA (CMPCPP and ATP are substates of 5'- and 3'-ends of the 2-mer RNA) base pairs with template DNA at the -1G and +1T positions, respectively, and the DNA/RNA hybrid is positioned at the post-translocated stage. Thus, in this complex, the +1T and +2G template DNA nucleotides are positioned at the *i* and *i+1* register of the RNAP active site, respectively. The open DNA bubble in this complex is akin to the RPo determined it this study. Basically, it is the RPo with a 2-mer RNA transcript base pairing with the -1 and +1 DNA bases. Please note that CMPCPP can be incorporated in RNA at 5'-position due to its having 3'-OH group as regular NTPs.

To briefly explain about RPtic formation and its structure, we modified the text at lanes 82-88:
In the course of cryo-EM data processing, 3D classification revealed 2 distinct structures (Methods, ***SFigs. 2****), corresponding to the closed promoter complex (RPc) and the transcript initiation complex (RPtic) containing 2-mer RNA. In the RPtic, the 2-mer RNA transcript (5'-CpA-3') base-pairs with -1G and +1T in template DNA and is positioned in the post-translocated conformation. Please note that CMPCPP can be incorporated into RNA at 5'-end due to having 3'-OH group as regular NTPs. The detailed RPtic structure will be described in a separate report.*

Currently, we are investigating the mechanisms of transcript initiation and promoter escape on rrnB P1 by determining several cryo-EM structures of the initiation complex and we will report the RPtic structure in a separate manuscript.

2. Page 3, line 76 and Page 5, line 133, the author included ATP/CMPCPP for sample preparation, does the map of Class 2 show the presence of the two initiating nucleotides? The previous literature claimed that the high concentration of initiating NTPs is critical for stabilizing rrBP1-RPo [PMID: 9405339], however, it looks that the scrunched rrnBP1-RPo could still be properly formed based on the structure. Does the conclusion still stand?

We agree with this reviewer's comment that high concentration of iNTPs can stabilize the *rrnB*P1 open complex, as shown by the R. Gourse group. We therefore prepared the RNAP and *rrnB*P1 promoter complex for cryo-EM both in the absence and in the presence of ATP and CMPCPP as iNTPs and determined both structures. Surprisingly, we captured the RPo structure showing the completely open bubble with scrunched DNA in the absence of iNTPs, as reported for RPo in this study, suggesting that this complex can form even in the absence of iNTPs.
We therefore plan to investigate the iNTP dependent DNA opening and its stabilization using the Cy3-dye incorporated into DNA oligos as reported by the Heyduck's group (DOI: 10.1042/bj20140179) and report the results in a separate manuscript.

3. Page 4, the second paragraph, it looks like the UP element-αCTD interaction is different from that in the rpsTP2-RPc structure reported by the Darst's group [PMID: 32160514]. It would be informative to add a supplemental figure for a detailed comparison of the UP element-αCTD interaction in the two structures.

We compared the DNA and αCTD interactions found in this study and the published structure of rpsTP2-RPc and added a new SI Figure (SFig. 5B) and modified the text (lanes 120-124) to explain different modes of the DNA and αCTD interaction in the absence and presence of the UP element:
The position and orientation of αCTDs in the rrnBP1 RPc structure is distinct from those of αCTDs in the RNAP complex with the rpsTP2 promoter, lacking the UP element (PDB: 6PSQ)[14]*, which binds DNA just upstream of the σ domain 4 (σ_4) bound at the -35 element (SFig. 5B). This indicates that the mode of αCTDs interactions with upstream DNA can be significantly different in various promoters depending on the presence of UP element.*

4. Page 4, line 100, although current RPc structure shows no obvious DNA bending upon

interaction with the αCTD dimer, however, the interaction of αCTD with a UP element further upstream might induce a significant bending of promoter DNA.

We agree with this reviewer's comment that RNAP interaction with the UP element and further upstream DNA may be required for the upstream DNA warring around RNAP. To clarify our observations obtained from the RPc structure with shorter upstream DNA, we modified the text (lanes 114-119):

However, αCTDs do not bend the DNA around their binding site in the RPc structure determined in this study. This observation suggests that the contacts of αCTDs with the UP element by themselves are not sufficient for wrapping of upstream DNA around RNAP. This may possibly result from destabilization of upstream DNA and RNAP interactions by truncation of the promoter fragment at the -60 position and/or from specific conditions used for the cryo-EM sample preparation.

5. Page 5, line 117, does the map support unambiguous modeling of the scrunched template and non-template ssDNA of the transcription bubble? The authors should provide an additional figure with a local map and fitted model to support the scrunched conformation of template strand DNA. Moreover, how is the scrunched nontemplate ssDNA accommodated?

Thank you for asking. The cryo-EM density supports unambiguous modeling of the template DNA within the open bubble (SFIg. 4B). We added a new SI Figure (SFig. 4E) to show the density map of the open bubble. Template DNA (the G-7 base) in the bubble fits well into a cavity between the σfinger and β'lid, enhancing its rigidity and making it visible in the density map. In contrast, the non-template DNA strand is more flexible, thereby bases from the -2 to +2 positions are disordered. We added the following sentence describing it in the text (lanes 175-177):

Non-template DNA is also scrunched, making DNA bases around single-stranded and double-stranded junction (from -2 to +2 positions) disordered.

6. Page 5, line 123, there should be a figure citation. The description is also confusing. Is the sR1.1 still present in the RPo complex?

To avoid confusion and clarify the interaction between the RNAP core enzyme and σ factor during the RPo formation, in the revised manuscript, we defined σ1.1 region until 91aa residue as used by Campbell *et al*, *Mol Cell* (2002) paper, and modified Fig. 2B and the text (lanes 148-152):

*The βgate loop (βGL, residues 368-378) in the βlobe domain contacts the T94, R99 and R103 residues of σ region 1.2 ($\sigma_{1.2}$, residues 92-127) to enclose the RNAP cleft (**Fig. 2B**). The βGL, $\sigma_{1.2}$ and $\sigma_{2.1}$ (residues 373-396) contact the ntDNA strand of the discriminator from positions -8 to -6 (**Fig. 2C, SMovie 3**); consistently, the βGL deletion and substitutions in $\sigma_{1.2}$ destabilize the RPo [26,27].*

7. Page 6, line 161, the author should provide a local cryo-EM map for the ppGpp and DksA.

We added a new SI Figure (SFig. 6E) to show the local maps for ppGpp (sites 1 and 2) and DksA of the RP2-DksA/ppGpp complex.

8. Fig. 5B, how were the two structures superimposed? Is there any movement of βlobe/Si1? Is there a direct clash between the β' clamp-toe and σR1.1? If not, how does the rotation of the σNCR induce clamp closure and subsequent σR1.1 ejection?

The structures are superposed using their αNTDs and the catalytic domains (DPBB domains from the β and β' subunits) as their references. We added corresponding description at the end of the "Structure refinement and analysis" section in Methods.
βlobe/Si1 moves slightly between the apo-form and RPc RNAPs but this motion is much less pronounced compared with the DksA/ppGpp induced motion shown in Fig. 5A. As described in the text (labes 244-248) and also shown in Fig. 5B, the rotation of σNCR closes the clamp domain and results in its clash with σ1.1 (shown as white oval), which triggers the σ1.1 ejection from the RNAP cleft:
*In the case of RPc, the $\sigma_{1.2}/\sigma_{NCR}$ rotation disrupts the βGL and σ contact and widens the gap that allows the ejection of $\sigma_{1.1}$ and the entrance of discriminator DNA for the open complex bubble formation (**RPc, Fig. 5A**). Compared with the apo-form RNAP, the σ_{NCR} and β'CT interaction in the RPc closes the β'clamp, which likely stimulates the ejection and prevents re-entry of $\sigma_{1.1}$ due to its steric clash with the β' clamp (**Fig. 5B**).*

9. Page 7, line 173, was the interaction of βlobe-SI1/DksA-CT also observed in the previous crystal structure of DksA-RNAP holoenzyme [PMID:29478808]? Were the DksA/ppGpp-induced conformational changes in βlobe/Si1, β'jaw/Si3 and β'clamp also observed in the previous crystal structure of DksA-RNAP holoenzyme?

The interaction of the βlobe-Si1/DksA-CT and the conformational change of βlobe-Si1 were also observed in the previous crystal structure of RNAP-DksA/ppGpp complex but not to the same extent as in this cryo-EM structural study. The interaction and the motion are smaller in the previous crystal structure likely due to crystal packing of RNAP that limits the motion of βlobe-Si1 upon the binding of DksA/ppGpp as described in the text (lanes 213-218):
*The interactions of the βlobe SI1 domain with DksA CT-helix and the conformational change of SI1 were previously observed in the crystal structure of the RNAP-DksA/ppGpp complex but were smaller than in the cryo-EM structure, likely because of the crystal packing. The conformational change in βlobe/Si1 establishes a new contact with the DksA CT-helix, which is only observed in the cryo-EM structure (**Fig. 4C, SMovie 4**); the deletion of βSi1 was shown to reduce the DksA affinity to RNAP and impair its function [36].*

10. Page 7 and Fig. 3, the model of σR1.2/NCR rotation triggered by σAL/promoter charge-repulsion is attractive. As proposed by the authors in Fig. 6, the ejection of σR1.1 requires rotation of σR1.2/NCR module, therefore, a much larger effect than observed here on RPo formation or RPo stability of σAL deletion would be expected. Have the authors analyzed the σNCR deletion mutant or RNAP-β'CT deletion mutant?

Thank you for suggesting these important experiments. We plan to generate these derivatives, analyze their effects on transcription initiation including promoter binding, DNA opening and also promoter escape, and report the results in a separate manuscript describing the mechanism of initiation of RNA synthesis on the rRNA promoter. Unfortunately, the current situation does

not allow to rapidly proceed with these experiments, so we would prefer to not include them in this manuscript.

11. Page 13, line 418. There should be two batches of sample preparation for RNAP-rrnBP1 promoter complexes but the text only describes one of them.

We added a detailed description for preparation of all promoter complexes as requested. Thank you for your suggestion.

12. Page 15, line 453, the defocus range should be "-1.0 to -3.0", also in the STable 1.

Corrected. Thank you.

Reviewer #2 (Remarks to the Author):

Review:
This very significant manuscript describes 4 moderate resolution (about 4 A) cryoEM structures of complexes of E. coli RNA polymerase and ribosomal (rrnBP1) promoter DNA (-60 to +20).

One complex, formed in the presence of the initiating nucleotide (ATP) and also the -1,-2 dinucleotide (CMPCPP) exhibits scrunched discriminator open strands and a correctly-positioned, open start site for initiation, presumably with bound nucleotides though these apparently are not observable in the structure. Another complex, apparently obtained without nucleotides using the same or similar conditions, is a closed complex which binds duplex DNA to +3. In this closed complex, the two alpha CTD interact with a subregion of the UP element (near -50). This complex, prepared using a 1:2 ratio of RNA polymerase to promoter DNA, has a second DNA molecule bound in the cleft. These structures are a significant addition to those previously available and provide new insights into initiation. But it seems inappropriate to call these structures RPo and RPc, as indicated below.

Two complexes with bound DksA and ppGpp (called RP1-DksA/ppGpp and RP2-DksA-ppGpp) are characterized, revealing aspects of how these factors work.

RP1-DksA/ppGpp shows DksA blocking the secondary channel to prevent the entry of NTP. It also shows a contact between DksA and the beta lobe, proposed to induce the conformational change involving beta lobe/Si1, beta' jaw/Si3 and beta' clamp that destabilize the open complex.

RP1-DksA/ppGpp has no detectable downstream DNA density, but the downstream DNA (+3 to +20) is observable in RP2-DksA/ppGpp. The -10 element is open while the discriminator and start site DNA is not observable in in RP2-DksA/ppGpp. The contact between DksA and beta lobe/Si1 is broken, indicating to the authors that the conformation transition between RP1 and RP2 may cause the dissociation of DksA and allow subsequent formation of the initiation complex.

DksA and ppGpp also partially open the DNA loading gate by moving the betalobe/SI1, but not make the ejection of sigma 1.1.

These structures also reveal new information about how sigma region 2 triggers the loading of DNA in the clamp and cleft. From comparison of their -DksA complex structures with that of holoenzyme they deduce that contact of duplex -10 element with sigma region 2 triggers a rotation of the large sigma non-conserved region (NCR) and movement of sigma region 1.2, disrupting the interaction of sigma with the beta gateloop (GL) and allowing the discriminator to enter the RNAP cleft. Unwinding of the -10 element then makes the sigma NCR rotate back to stabilize the open complex.

Comments/Questions:

1) It seems more appropriate to call the open complex RPitc and not RPo, given that it is formed in the presence of NTP and presumably would not be stable enough to observe without bound NTP at +1 and -1, -2.

In this study, we prepared the RNAP-rrnBP1 complex with and without iNTPs (ATP and CMPCPP). Unexpectedly, in the presence of iNTPs we detected formation of RPtic (we prefer to call this complex as transcript initiation complex, RPtic instead of RPitc as indicated by this reviewer) and RPc (SFig. 2), whereas conditions without iNTPs resulted in the formation of RPo (SFig. 3). The RPtic indeed contains *de novo* synthesized 2-mer RNA as this reviewer described.

2) While the text of the manuscript (line 75) seems clear that the closed complex was obtained without nucleotides, the following sentence would then refer to the conditions for preparing the open complex, but is in the section on the closed complex and could be interpreted as meaning a second attempt to make a closed complex. The methods and captions to the SI figures are also ambiguous on this point. In all places, clarification is needed.

Thank you for your suggestion. We have modified the text (lanes 82-88, lanes 500-506) and SI Figures to clarify the details of our sample preparations and structure determinations.
In the course of cryo-EM data processing, 3D classification revealed 2 distinct structures (Methods, ***SFigs. 2****), corresponding to the closed promoter complex (RPc) and the transcript initiation complex (RPtic) containing 2-mer RNA. In the RPtic, the 2-mer RNA transcript (5'-CpA-3') base-pairs with -1G and +1T in template DNA and is positioned in the post-translocated conformation. Please note that CMPCPP can be incorporated into RNA at 5'-end due to having 3'-OH group as regular NTPs. The detailed RPtic structure will be described in a separate report.*

To prepare the RNAP and rrnBP1 promoter complex, E. coli σ^{70} RNAP (20 µM) and rrnBP1 promoter DNA (40 µM) were preincubated for 5 min at 37 °C in buffer (10 mM HEPES, pH 8.0, 50 mM NaCl, 0.1 mM EDTA, 5 mM DTT and 5 mM $MgCl_2$). This sample was used for determining the RPo cryo-EM structure. iNTPs (ATP and a nonhydrolyzable CMPCPP (cytidine-5'-[(α,β)-methyleno]triphosphate, Jena Bioscience) (2 mM each) were added to the RNAP-rrnBP1 promoter complex to stabilize the RPo, but this condition formed the RPc and RPtic as described in the Results.

Good evidence exists for a series of at least four closed complex intermediates which differ greatly in structure but are very similar in stability on the pathway to open complex formation (see Sreenivasan et al, Biochemistry '20 and refs therein). In all but the initial closed complex, the upstream promoter DNA is found by fluorescence to be highly bent and wrapped (to about -82). The closed complex characterized in the manuscript under review appears different from all closed complexes studied previously, with downstream characteristics like those of a more advanced closed complex and upstream characteristics more like those of the initial closed complex (traditionally called RPc). This may result from destabilization of upstream interactions by truncation at -60 or from perturbations from the second bound DNA and/or the cryoEM sample preparation. It seems important to state the above points explicitly.

The authors do not observe bending and wrapping of the upstream DNA in their open complex, also formed using DNA truncated at -60, whereas AFM, FRET, single molecule force and backbone footprinting all provide strong evidence for upstream wrapping of promoter DNA using a fragment extending to or beyond -100. Hence the absence of upstream wrapping in their closed complex isn't surprising. Since the authors make a point of the lack of upstream bending and wrapping in their closed complex, they should make this comparison for the open complex as well. In doing so it seems worth saying that the lack of upstream wrapping in their open complex could result from destabilization of upstream interactions by truncation at -60, or from perturbations of wrapping by binding the initiating NTP, or from the cryoEM sample preparation.

Thank you for your suggestion. We have indicated this in the revised manuscript (lines 114-119): *However, αCTDs do not bend the DNA around their binding site in the RPc structure determined in this study. This observation suggests that the contacts of αCTDs with the UP element by themselves are not sufficient for wrapping of upstream DNA around RNAP. This may possibly result from destabilization of upstream DNA and RNAP interactions by truncation of the promoter fragment at the -60 position and/or from specific conditions used for the cryo-EM sample preparation.*

RPc is the designation of the initial closed complex in which the downstream duplex is not strongly bent and makes contacts with RNA polymerase only to about -5, and in which the upstream DNA is also not strongly bent or wrapped. Perhaps the closed complex studied by the authors could be designated RPcc, since it appears quite different than any studied previously.

The RPc structure that is described in this manuscript is in fact very similar to the definition RPc proposed by the reviewer, since its upstream DNA is not strongly bent and the downstream duplex is not strongly bent as well, and RNAP makes contacts with DNA only to about -5 (Fig. 1B). We also note that the structure of RPc presented here is the first structure of the closed promoter complex obtained in the absence of cofactors, and the first structure for the rrnBP1 promoter (for which much less biophysical evidence on DNA melting is available).
We also thank you for your suggestion to use longer DNA for further structural analysis. We will test a longer DNA fragment in the future to observe DNA bending on both upstream and downstream parts of the complex.

3) Revisions are needed in lines 45-47 and 148-9. Both talk about facilitation of rrnBP1-promoter escape by reducing the abortive RNA cycle prior to the productive RNA elongation

stage, but Shimamoto, Record and others have found for lambdaPr, T7A1 and lacCONS promoters that abortive initiation is performed only by a nonproductive subclass (about 50 %) of initiation complexes which are incapable of promoter escape, and not by productive initiation complexes. For other promoters, Heyduk, Record and others observed correlations between the stability of the open complex and rate of escape and the hybrid length for escape which may be relevant for rrnBP1.

Growing evidences suggest that each promoter has its unique properties for determining the kinetics of promoter escape. As this reviewer described, several promoters form non-productive RNAP and DNA complex that produce the majority of abortive products. However, in rrnBP1 the formation of such non-productive complex is minimal, ensuring high expression of rRNA during the log-phase (Winkelman et al., 2016 PNAS). The absence of the abortive initiation cycle may therefore ensure both the higher rate of promoter escape and the lower fraction of non-productive complexes formed during initiation for this promoter, as we indicate in the revised manuscript (lanes 183-186) and cited papers from the Shimamoto and the Record groups:
Since the σ finger is the major obstacle to promoter escape [31-33], the partially displaced σ finger in the rRNA promoter RPo due to open complex scrunching may reduce abortive RNA cycle or may prevent formation of inactive moribund complexes[30,34], promoting the robust expression of rRNA.

However, we would like to note that promoter escape is not the main research topic in this manuscript. We are currently investigating the structural mechanism of promoter escape from *rrnB* P1 by analyzing several transcription inhiation complexes by cryo-EM and plan to cite all relevant literature on this topic (including studies from Shimamoto, Record and Heyduk groups) in the future manuscript reporting the structural basis of promoter escape.

4) Lines 111-2. The authors say sigma region 2.3 fits into the major groove of the duplex DNA in their closed complex, but that there are no specific contacts between amino acids and DNA bases. Can they be sure of this, given their resolution? Could this lack of specific interaction with the -10 region be caused by the presence of the second bound DNA molecule? Are base specific interactions observed with the -35 region and the UP element, or are these interactions similar to those observed for the -10 element?

Although the RPc structure was not determined at atomic resolution, it is clear enough to position both σ2.3 and the -10 element DNA, allowing us to investigate that there is no sequence specific interaction between the σ2.3 and -10 element DNA bases. The second DNA positions away from the dsDNA (-10 element) on σ2.3 so it doesn't directly influence the -10 element and σ2.3 interaction. However, it can indeed be possible that the second DNA binding may 'freeze' the RPc conformation, not allowing it to proceed for further DNA bending and melting. However, as we described in the manuscript, the Darst group characterized the interaction between the σ2.3 and dsDNA form the -10 element and reported that the σ2.3 does not directly recognize the DNA sequence of the -10 element in the dsDNA form, confirming that the RPc complex represents a natural intermediate in the open complex formation pathway.
Region σ4.2 recognizes the -35 element DNA using classical helix-turn-helix motif whereas αCTDs recognize the UP element by its shape recognition as reported by a recent paper from the Catherine Lawson's group (the manuscript is under review by Biochemistry). The -35 element

and σ4.2 interaction has been published and our structures do not provide any new insight about it, so we do not describe anything about the -35 and σ4.2 contacts in this manuscript. In contrast, our study shows for the first time αCTD binding to the UP element of rRNA, and we therefore describe its mode of binding in more detail and cite the study of the Lawson's group (lines 103-106):

The αCTDs bind DNA with a narrow minor groove, which is formed due to the presence of an A/T stretch sequence, as revealed by the recent X-ray crystallographic study of the αCTD and UP element interaction (C. Lawson, personal communication).

5) Figure 3 caption and/or the accompanying text should clarify that the determinations of lifetime of open complexes and stability of DksA interaction are performed with negatively supercoiled DNA. The caption and SI methods should also describe the methods used to determine lifetime/stability and the use of heparin vs. fork junction competitor.

Thank you for this comment. In the revised manuscript, we indicated both in Methods and in the Figure 3 caption that the experiments were performed with negatively supercoiled DNA. We also noted an error in our description of the experiments in the figure caption. All lifetime/stability experiments were performed with the fork-junction DNA as a competitor, as described in detail in materials and methods. The caption erroneously said that heparin was used instead. We corrected this in the revised manuscript.

6) Lines 237-9 and other places. In their remarks regarding the -DksA mechanism for open complex formation it would be appropriate to refer to recent structural and solution studies of this mechanism from Darst, Record and other laboratories.

We referred to the recent structural study from the Darst group in several placed already (page 3, lanes 65-67; page 6, lanes 145-147; page 8, lanes 216-219; page 9, lanes 246-248).
We also introduced studies of the mechanism of open complex formation from the Record's, Heyduck's and deHaseth's groups in Introduction.

Reviewer #3 (Remarks to the Author):

Regulation of ribosomal RNA (rRNA) transcription during stringent response is critical for bacteria adaptation and survival. The regulation is achieved on the level of transcription initiation on specific rRNA promoters and their interaction with RNA polymerase (RNAP). Moreover, the transcription factor DksA, which directly binds RNAP, drastically effects rRNA promoters activity by sensing the bacterial alarmone 'ppGpp'. There are two recently published studies revealing series of cryo-EM structures of a DksA homolog, TraR, dependent transcription system (Chen et al, eLife, 2019 and Chen et al, Moll Cell, 2020). However, the latter studies are conducted on different class of bacterial promoters.

In this manuscript, Shin et al reveal important new details specifically of the rRNA transcription regulation based on four cryo-EM structures of rRNA promoter/RNAP complexes. In detail, the authors describe the critical step of the initiation pathway, the formation of so called 'open complex' on rRNA promoter, with and without DksA/ppGpp present. By detail comparison of these alternative complexes, the authors propose two plausible pathways of the process. It also allows them to mechanistically describe the role of DksA/ppGpp in rRNA promoter regulation and transcription initiation. These are important advances towards the broad understanding of the rRNA transcription regulation during stringent response.

The added value of the manuscript is also the visualization of the RNAP αCTD subunits interaction with the UP element of DNA promoter. This interaction has been known, but only now characterized structurally.

The manuscript is written in a concise way and well illustrated by five very descriptive movies.

I recommend the manuscript for publication in Nature Communication after the following concerns have been addressed.

Thank you for reviewing our manuscript, recommending the manuscript for publication and providing many insightful comments for improving quality of this manuscript. We have addressed your critiques and comments as described below:

1. Line 48: The term 'optimized and nonoptimized' growth conditions is not very clear. What kind of optimisation do the authors mean?

Thank you for asking clarification. We have modified this sentence (lanes 48-50) as following:
These promoter elements play key roles in the wide range of rRNA transcription regulation between nutrient-rich and -poor growth conditions.

2. Line 63: The authors should explain the term 'stressed RPo'.

We have removed "stressed" from this sentence.

3. Line 94-95. The UP element has a conserved sequence. Can the authors explain why is it specifically bound by the αCTDs, while they don't report any sequence specific contacts? Is

there for example any specific shape of the DNA in UP element region, similarly to what is described for the -10 element?

αCTDs binds DNA with a narrow minor groove, which is formed when stretch of at least 6 A/T bases is present. A recent crystallographic and computational study done by the Lawson's group nicely explained the DNA sequence requirement to establish the αCTD binding site on DNA. In the revised manuscript, we have described this study at lanes 103-106:

The αCTDs bind DNA with a narrow minor groove, which is formed due to the presence of an A/T stretch sequence, as revealed by the recent X-ray crystallographic study of the αCTD and UP element interaction (C. Lawson, personal communication).

4. Line 137-138: The authors should clarify which promoter-RNAP complex the D256A mutation stabilizes. Do they mean stabilisation of the 'un-scrunched' -3A TSS RPo complex and destabilisation of the regular +1A TSS RPo. Could that be confirmed by the identity of the TSS in the transcription assays in Figure3A?

During revision, we have performed TSS mapping for promoter complexes formed by wild-type and mutant variants of *E. coli* RNAP analyzed in this study. We have demonstrated that the D256A mutation in the β subunit (and, similarly, deletions Δσ1.1 and ΔσAL in σ^{70}) do not affect the TSS selection by RNAP and that all RNAP variants preferentially initiate transcription at the regular position 9 bp downstream of the -10 element (+1A), with only minor initiation observed for -3A. These data are presented in the revised Figure 3D. We describe this in the revised manuscript as following (lines 170-177):

*However, the D256A substitution did not change the TSS in rrnBP1, as revealed by primer extension analysis of the transcription products synthesized from rrnBP1 by wild-type and mutant RNAPs (**Fig. 3D,** lanes 3 and 4). Therefore, the contacts of the β'lid with G-7 may decrease the stability of the RNAP-rrnBP1 complex with scrunched DNA, possibly by favoring the unstable conformation of the template strand, without changing the TSS selection.*

5. Line 198-199: The authors claim that ''The βGL contacts σ1.1 and σ1.2 to enclose the RNAP cleft in the apo-form RNAP (Fig. 5A) and that 'the same interaction' in RPo stabilises the open complex bubble (Fig. 2B)'. While there is σ1.1 clearly marked in Fig. 5A, it is not in Fig. 2B, and neither 2C. It also seems the σ1.1 is not ordered/visible in neither of the RPo complexes. The authors should clarify role of σ1.1 in open complex bubble stabilisation and clearly mark it in all mentioned figures.

To avoid confusion and clarify the interaction between the RNAP core enzyme and σ factor during the RPo formation, in the revised manuscript, we defined σ1.1 region until 91aa residue as used by Campbell *et al*, *Mol Cell* (2002) paper, and modified Fig. 2B and the text (lanes 148-152):

*The βgate loop (βGL, residues 368-378) in the βlobe domain contacts T94, R99 and R103 residues of σ region 1.2 ($\sigma_{1.2}$, residues 92-127) to enclose the RNAP cleft (**Fig. 2B**). The βGL, $\sigma_{1.2}$ and $\sigma_{2.1}$ (residues 373-396) contact the ntDNA strand of the discriminator from positions -8 to -6 (**Fig. 2C, SMovie 3**); consistently, the βGL deletion destabilizes the RPo and shifts the TSS to the -3A position [22].*

6. Line 218-219: The authors claim the TraR structures did not show σ1.2/σNCR rotation or σ1.1 ejection. In the late stages of TraR mediated RPo formation the σ1.1 is actually ejected (Chen et al., Moll Cell, 2020, Video S3). The authors should clearly specify which state of both systems they are comparing.

Thank you for asking clarification. We specifically compared the step of the closed complex formation for our complex with rrnBP1 and for the previously published complex with *rpsT*P2. To clearly specify the state of both systems, we modified the text (lanes 271-275) as following: *Similarly, the structures of the RNAP-TraR complex and several RNAP-DNA complex intermediates prepared in the presence of TraR also showed the opening of the DNA loading gate by shifting the βlobe/Si1 position but did not show the $\sigma_{1.2}/\sigma_{NCR}$ rotation. Furthermore, $\sigma_{1.1}$ was not ejected from the RNAP cleft at the stage of RPc formation (**SFig. 9A**)* [13,34].

7. Line 237-241: The authors describe the pathway of RPo formation without DksA/ppGpp. The first steps include ejection of σ1.1. This is different to what is proposed in Chen et al. (Moll Cell, 2020, Video S4). Can authors explain this discrepancy? Do the authors think the proposed pathway can still be only rRNA promoter dependent?

The reason why the previously proposed pathway is different to ours is that Chen et at. investigated RPo formation in the presence of the transcription regulator TraR, which is a functional analog of DksA/ppGpp. Their proposed pathway is consistent with the pathway of RPo formation in the presence of DksA/ppGpp proposed in this study. Chen et al. did not investigate the pathway of RPo formation without the bound transcription factor, probably because the closed and intermediate complexes with the *rpsT*P2 promoter could not be formed under their conditions.
We also cannot rule out the possibility that the RPo formation pathway proposed in this study is rRNA promoter specific since we haven't investigated the structures of complexes with non-rRNA promoter yet. We plan to investigate the RPo formation pathway with other well characterized promoters such as λ_{PR} and T7A1 to address this question in our future studies. We indicate in the text (lanes 293-297) that this pathway is based on our analysis of *rrnB*P1:
We hypothesize that RNAP may use alternative mechanisms of RPo formation with rrnB P1 and possibly other promoters, requiring opening of the DNA loading gate (disrupting the βGL contact to σ), $\sigma_{1.1}$ ejection from the DNA binding channel, and unwinding of the -10 element plus discriminator DNA, depending on the absence or presence of DksA/ppGpp.

8. The protein validation statistics in Table S1 reports high clash scores. Generally the clash scores in cryo-EM structures are higher then in X-ray crystallography, but the reported numbers higher then standard. The authors should carefully inspect these and try to correct it. Especially in low resolution regions, where the cryo-em density probably does not define the model so well. The authors should also report cross-correlation of the atomic model with the cryo-EM map.

Thank you for your advice. We refined all models to reduce their clash scores substantially (from 48~62 to 14~18) and deposited revised coordinates in PDB. We report CC in Table S1 as suggested.

9. It will probably not change the overall interpretation of the cryo-EM structure analysis, however the authors could try to improve the 3D cryo-EM reconstructions by i) per particle CTF fitting, ii) refinement of optical aberrations and iii) Bayesian polishing and limiting the total fluence to ~26-30 e-/Å2 (Grant, eLife, 2015).

Thank you for your advice. Truncating a few initial and final frames of the movie stacks to limit the total dose and enhance the signal-to-noise ratio (SNR) is counterintuitive when Bayesian polishing is performed. The B-factor weighting implemented by polishing automatically improves the SNR. We used all the frames as we ought to polish the particles.
We have performed per-particle CTF refinement and Bayesian polishing for one dataset but the improvement (0.1 Å) was not significant and came at the cost of time and computational resources at our disposal. Therefore, we opted not to run this additional processing step in our pipeline.

Reviewer #1 (Remarks to the Author):

The authors have addressed all of my concerns. A minor typo correction.
Page 10, line 261, 'case' should be 'cause'.

Reviewer #2 (Remarks to the Author):

Revisions made in response to the reviewers' comments have greatly improved the clarity of the manuscript and the impact of this very significant research. The authors cover letter shows the very commendable consideration they gave to all these comments, whether or not they agreed with them.

Two recommendations for minor revision:

In line 84 and subsequent references to their closed complex structure, this reviewer recommends the following wording ....corresponding to a closed promoter complex (here designated RPc).... The reason for this is that there is very solid foot printing evidence from the 1980's for two forms of the closed complex (called RPc1, RPc2) with very different downstream footprint boundaries. And recent fluorescence kinetic-mechanistic studies (cited as ref 15) have obtained strong FRET structural evidence for four different closed complexes differing in the position of both the downstream and the upstream DNA. In these previous studies, RPc (or RPc1) is is the initial closed complex, but it is not likely (as stated in my previous review) that the closed complex visualized by cryoEM in this research is the same as that initial RPc. Instead the authors closed complex is a more advanced closed complex with the downstream DNA bent onto RNAP (designated as an I1 species in ref 15), which would have an extended downstream footprint as in RPc2.

In line 261, almost certainly the authors mean cause, not case.

Reviewer #3 (Remarks to the Author):

The authors have addressed all my remarks and comments satisfactorily. Moreover, they extended their analysis to for additional experiments (Fig. 3D) to strengthen the manuscript.

From my point of view, the manuscript is suitable for publication.

Reviewer #1 (Remarks to the Author):

The authors have addressed all of my concerns. A minor typo correction.
Page 10, line 261, ‘case’ should be ‘cause’.

Corrected. Thank you

Reviewer #2 (Remarks to the Author):

Revisions made in response to the reviewers' comments have greatly improved the clarity of the manuscript and the impact of this very significant research. The authors cover letter shows the very commendable consideration they gave to all these comments, whether or not they agreed with them.

Two recommendations for minor revision:

In line 84 and subsequent references to their closed complex structure, this reviewer recommends the following wording ....corresponding to a closed promoter complex (here designated RPc).... The reason for this is that there is very solid foot printing evidence from the 1980's for two forms of the closed complex (called RPc1, RPc2) with very different downstream footprint boundaries. And recent fluorescence kinetic-mechanistic studies (cited as ref 15) have obtained strong FRET structural evidence for four different closed complexes differing in the position of both the downstream and the upstream DNA. In these previous studies, RPc (or RPc1) is the initial closed complex, but it is not likely (as stated in my previous review) that the closed complex visualized by cryoEM in this research is the same as that initial RPc. Instead the authors closed complex is a more advanced closed complex with the downstream DNA bent onto RNAP (designated as an I1 species in ref 15), which would have an extended downstream footprint as in RPc2.

Thank you for your suggestion. We modified the main text describing closed complex as suggested by this reviewer.

In line 261, almost certainly the authors mean cause, not case.

Corrected. Thank you.

Reviewer #3 (Remarks to the Author):

The authors have addressed all my remarks and comments satisfactorily. Moreover, they extended their analysis to for additional experiments (Fig. 3D) to strengthen the manuscript.

From my point of view, the manuscript is suitable for publication.
Thank you.
